# Uneven adaptive capacity among fishers in a sea of change

**DOI:** 10.1371/journal.pone.0178266

**Published:** 2017-06-12

**Authors:** Joshua S. Stoll, Emma Fuller, Beatrice I. Crona

**Affiliations:** 1 School of Marine Sciences, University of Maine, Orono, Maine, United States of America; 2 Maine Center for Coastal Fisheries, Stonington, Maine, United States of America; 3 Department of Ecology and Evolutionary Biology, Princeton University, Princeton, New Jersey, United States of America; 4 Global Economic Dynamics and the Biosphere, The Royal Swedish Academy of Sciences, Stockholm, Sweden; 5 Stockholm Resilience Center, Stockholm University, Stockholm, Sweden; Sveriges lantbruksuniversitet, SWEDEN

## Abstract

Fishers worldwide operate in an environment of uncertainty and constant change. Their ability to manage risk associated with such uncertainty and subsequently adapt to change is largely a function of individual circumstances, including their access to different fisheries. However, explicit attention to the heterogeneity of fishers’ connections to fisheries at the level of the individual has been largely ignored. We illustrate the ubiquitous nature of these connections by constructing a typology of commercial fishers in the state of Maine based on the different fisheries that fishers rely on to sustain their livelihoods and find that there are over 600 combinations. We evaluate the adaptive potential of each strategy, using a set of attributes identified by fisheries experts in the state, and find that only 12% of fishers can be classified as being well positioned to adapt in the face of changing socioeconomic and ecological conditions. Sensitivity to the uneven and heterogeneous capacity of fishers to manage risk and adapt to change is critical to devising effective management strategies that broadly support fishers. This will require greater attention to the social-ecological connectivity of fishers across different jurisdictions.

## Introduction

Change is often considered the only unconditional constant in natural resource based sectors, including commercial fisheries. This perpetual flux is influenced by the biophysical and ecological characteristics of the environment [1] and socioeconomic pressures such as shifts in market demand and consumer preferences [2]. Each of these many forces interact at multiple and simultaneous scales making it difficult to anticipate the direction, pace, or implications of change—only that it will happen [3–5]. Those who are nimble and can leverage the resources necessary to adapt their strategies are generally thought to be better suited to withstand change than those who are locked into narrowly defined arrangements [6,7]. While acknowledgement of the role that adaptive capacity plays in sustaining both fishers and fisheries has increased over time [8–11], attentiveness to the complex and diverse connections that fishers have to different fisheries, often across multiple jurisdictions, has been largely missing. In this article we bring explicit attention to the heterogeneity of fishing strategies employed by fishers by characterizing the different assemblages of fisheries that fishers harvest and evaluating the extent to which individuals’ ability to navigate change differs as a result.

Fishers worldwide employ a wide range of strategies to ameliorate the potential impacts caused by socioeconomic and ecological changes. One of the commonly referenced approaches involves maintaining access to diverse fishing portfolios of uncorrelated or disassociated species [5,12,13]. Being able to fish for diverse sets of fisheries is typically achieved by holding access privileges to multiple species through permits, licenses, quota, or territorial use rights that are acquired from various governing bodies or purchased or leased from private firms. This diversification gives fishers the ability to shift between fisheries based on what is most convenient, abundant, or valuable at a particular moment in time. The advantage of this strategy is that fishers have multiple pathways through which they can maintain their livelihoods, whereby reducing income variability over time that might be caused by regulatory closures, over harvesting, or market shifts. Having this mobility also allows fishers to temporarily reduce fishing pressure on a species in a particular area to accelerate recovery and rebuilding [14] and it can help fishers accumulate wealth by enabling them to capitalize on valuable fisheries during periods of abundance [15].

Though diversification represents an important adaptation strategy, in practice, fishers’ ability to broaden their fishing opportunities is becoming increasingly difficult. In many places, fishers are becoming more reliant on an ever narrower suite of fisheries [16,17]. This trend can be attributed, in part, to the nature of the rules and regulations that are being implemented to curb overfishing and sustain fish stocks. These rules not only act to constrain the types of gear used, where and when fish are targeted, and the size of fish that can be taken, but they also act to constrain who and how many people can participate by way of limiting entry. In Maine, for instance, many of the most valuable fisheries have become limited entry, including those for elver, lobster, sea cucumber, alewife, and softshell clam. These restrictions afford fishers a level of security around which they can plan their businesses and make decisions about how and when to invest in these fisheries, but they also reduce flexibility, so that fishers that do not already hold the prerequisite permits or quota cannot easily shift to different fisheries or expand their fishing portfolios.

The risk associated with being over-reliant on a particular fishery or set of fisheries is not new. However, it is arguably exacerbated by the rapid pace of change that is underway. From an ecological perspective, changing global conditions are catalyzing unprecedented shifts in ocean and coastal conditions. In the Gulf of Maine, sea surface temperatures have been increasing rapidly over the past three decades, impacting many of the most economically important commercial fisheries in the region, including cod and lobster [18]. Major socioeconomic change is also taking place as seafood trade becomes increasingly globalized. One of the most apparent changes is the recent rapid expansion of seafood trade to China [19,20]. In 2015, $90.1 million worth of lobster alone was exported to China [21].

The magnitude of this change combined with increasing regulatory inflexibility raises important questions and also concern about the capacity of fishers to adapt. This perspective is reflected in numerous policy documents that specifically call for management actions that facilitate flexibility, mobility, and adaptation such as the US National Ocean Policy and the NOAA Fisheries Climate Science Strategy. To begin to understand the adaptive capacity of fishers in earnest requires greater attention to the numerous and diverse connections that marine resource harvesters have to different fisheries and how this diversity affects their business decisions and the resulting and pressure on fisheries. These differences will directly affect individuals’ ability to sustain their livelihoods and respond to changing conditions. Focusing on these connections at the level of the individual is especially critical (but uncommon) because ultimately adaptation occurs at this scale and not at the level of the community in the short-term.

In this paper we use the case of Maine to describe the uneven capacity of the commercial fishing sector to adapt to changing socioeconomic and environmental conditions based on fishers’ different connections to state and federal fisheries. Maine is among the most fisheries dependent states in the US and can thus be considered an interesting example and microcosm for the study of fishers-fisheries relations, which presumably exist in fisheries worldwide, but are seldom explicitly acknowledged. In demonstrating the link between the diversity of individual fishing portfolios and adaptive capacity in Maine we aim to highlight a dynamic that is relevant to fisheries across the globe. We start by (1) identifying the connections that fishers have to different state and federal fisheries. We then (2) characterize the commercial fishing fleet in Maine by creating a typology of fishers based on the fishing licenses that individuals hold. Lastly, we (3) evaluate the adaptive capacity of each type of fisher in the typology. This final assessment is based on a set of attributes and associated criteria identified by fisheries experts with extensive knowledge of Maine’s commercial fishing sector, including fishermen themselves. Our analysis reveals significant heterogeneity among fishers in their adaptive capacity based on their fishing portfolios, suggesting that the impacts of impending change in the Gulf of Maine will be highly uneven. Lack of attention to this heterogeneity at the individual level may result in overly blunt policy strategies that produce ineffective policies and subsequent management responses.

## Methods

### Connectivity across fisheries

To measure the connectivity of fishers to different assemblages of fisheries we created an association network based on the state and federal licenses that fishers held in Maine during 2014. Data used in this study belong to the Maine Department of Marine Resources and the National Marine Fisheries Service. These data can be requested from the Maine Department of Marine Resources' Licensing Division (http://www.maine.gov/dmr/commercial-fishing/licenses/index.html). Federal permit data is available online and can be obtained through the National Marine Fisheries Service Greater Atlantic Region Permit Office (https://www.greateratlantic.fisheries.noaa.gov/aps/permits/). This analysis excluded licenses for aquaculture leases, tribal fisheries, scientific sample collecting, transporting or distributing seafood, and recreational fishing. Edges in the network (i.e., links) represent linkages between pairs of fisheries in which a minimum of one fisher holds both licenses. Freeman’s degree centrality was calculated as a proxy for the relative importance of fisheries in the network [22].

### Types of fishers

We used a multiple correspondence analysis (MCA) to populate a matrix of Euclidean distances between license types. These data were used to conduct an agglomerative Hierarchical Cluster Analysis (HCA) using the R package FactoMineR. Using this approach, clusters were parsed apart to maximize the difference in the total variance within and between groups as described by Husson et al. [23]. We first performed this analysis using all the licensing data to form first order clusters in the typology that provided the basis for a second HCA, which in turn resulted in second order clusters that were then used for third, fourth, and fifth order clusters. In taking this approach, we were able to create a set of nested clusters that became progressively more homogenous as they were further subdivided with each subsequent HCA.

### Heterogeneity within the fishing fleet

We developed an index to describe the adaptive capacity of the commercial fishing fleet in Maine based on six attributes associated with adaptability in fisheries (S2 Table). These attributes and the decision criteria for each of them were established based on input from 13 individuals with expertise in fisheries in Maine using a modified Delphi process. The interviews associated with this process were approved by the University of Maine’s Protection of Human Subjects Review Board (Project # 2016-03-20). The Delphi method is an approach used to facilitate structured communication about complex concepts among experts [24]. This iterative process of triangulation involves individual feedback on a set of information followed by revisions and subsequent refinement with the aim of working towards consensus. The approach is predicated on the view that expert judgment needs to be calibrated because experts have different levels of training, research, and direct hands-on experience and these differences directly affect their understanding and orientation to ideas and information. Using this approach, experts were interviewed about the factors that make fishers adaptable, starting with an initial set of attributes that were based on a literature review of factors related to fishers’ adaptive capacity. Interviews were transcribed and coded thematically. The outcome of this first round of interviews was a preliminary set of attributes and associated criteria corresponding to a measure of adaptability (i.e., high–low). These preliminary attributes and criteria were shared back with interviewees and everyone was given an opportunity to respond. Based on this feedback, a final set of attributes and criteria were developed (Table 1). The criteria were then used to assess all state and federal fisheries, such that each fishery or combination of fisheries associated with a license received a score. Each license or combination of licenses was then evaluated to calculate an index for the typology. We emphasize that the attributes and subsequent decision criteria developed through this process are not necessarily the only factors that can affect adaptability. Therefore, any interpretation of these results needs to be sensitive to our approach to defining adaptability.

**Table 1:** Adaptive capacity criteria.

Attribute	Description	Metric
**1. Market stability**	Stable markets reduce fishers’ economic uncertainty in an otherwise dynamic and often unpredictable business environment.	We use the R^2^ value of the temporal trend in ex-vessel prices during the past 20 years (1995–2014) as a proxy for market stability. Price data ($) were provided by the Maine Department of Marine Resources.
**2. Status of fishery**	Healthy fish stocks lead to fishing opportunities.	We use stock assessment data from agency documents to determine the status of fisheries. In instances where stock assessments are not available we refer to recent research. Where no stock assessment data are available, we use the direction (-/+) of the temporal trend in landings over the past 5 and 20 years as a proxy for the health of each fishery.
**3. Potential to accumulate wealth**	Savings create a buffer that allow people to withstand lean periods.	We use the average value ($) of the fishery over the past 5 years divided by the number of harvesters with access to the fishery. For fisheries that are harvested with state and federal licenses, this value was weighted by the percentage of landings per sector.
**4. Existing local governance structure and industry organization(s)**	Participatory governance structures and self-organized fisheries organizations increase marine harvesters' ability to influence the system.	We measure governance and industry organization as the presence/absence of (1) a regional management structure with local stakeholder engagement; and (2) fisheries-specific associations.
**5. Geography**	License portfolios that give harvesters access to a diverse set of geographies increase marine harvesters' flexibility.	We define geographic diversity of a portfolio as the number of general regions (intertidal, state, and/or federal) that a harvester can access with his/her license portfolio.
**6. Gear type**	Using different gear types allows harvesters to target a more diverse set of fisheries and increases marine harvesters' opportunities to take advantage of fisheries more consistently.	We define gear diversity of a portfolio as the number of general scales of operation (hand/rake/net, hook/trap/dive, trawl/dredge/seine) that a harvester uses to exploit a particular fishery.

Attributes used to evaluate adaptive capacity of Maine’s commercial fishing fleet. See S2 Table for further detail.

The aim of the Delphi method is to establish a consensus opinion among experts. This is difficult to accomplish, however, given the diverse perspectives that experts bring to complex issues such as fisheries. Acknowledging this issue, the attributes used in the index are based on majority opinion and do not signify a complete consensus. These majority opinions are summarized in Table 1 (See “Description”) and illustrated further by way of quotes from interviewees in S2 Table. We also include quotes from interviewees that highlight dissenting views.

Attributes 1–4 were assessed for each of the state and federal species landed in Maine based on the metric defined in Table 1. These metrics provided a mechanism to systematically evaluate the attributes through a transparent and replicable process. Based on these metrics, each species was ranked 1 to 3 using the criteria defined in S2 Table. Where no data were available for a particular attribute, the fishery did not receive a score. In instances when more than one fishery was associated with a particular license (e.g., the general category commercial fishing license), scores were weighted based on the relative proportion of species captured in the portfolio. Species that can be landed with either a state or federal license (e.g., spiny dogfish) were also weighted based on the relative percentage of landings per sector. These scores were then applied to each group identified in the typology using the following logic:

If the marine resource(s) associated with a license is present in Gulf of Maine, then sum scores from attributes 1–4 scores. If the marine resource(s) is not present, then score as 0. Each species receives 1 point for attribute 5 and 6. Individuals with licenses that are based in different places (attribute 5) and/or require different gear (attribute 6) can receive up to 2 additional points per attribute.

Groups received scores for each license in their fishing portfolios. For example, a group with three unique licenses received three scores reflecting the sum of Attributes 1–4 for each license type. The highest possible score a single license type could receive was 12. Groups also were scored based on Attributes 5 and 6. The combined score for Attributes 5 and 6 ranged from 2 to 6. These scores were based on geographic and gear type diversity as described in S2 Table. To create the Adaptability Index, the maximum score associated with each group in the typology was summed with the scores from Attributes 5 and 6. Taking the maximum score rather than an average ensured that marine harvesters that hold multiple licenses were not penalized for holding a license to a “lesser” fishery. Raw scores were then scaled to 100. We intentionally did not weight the attributes used in the evaluation of adaptability.

## Results

### Connectivity across fisheries

**Fig 1:**
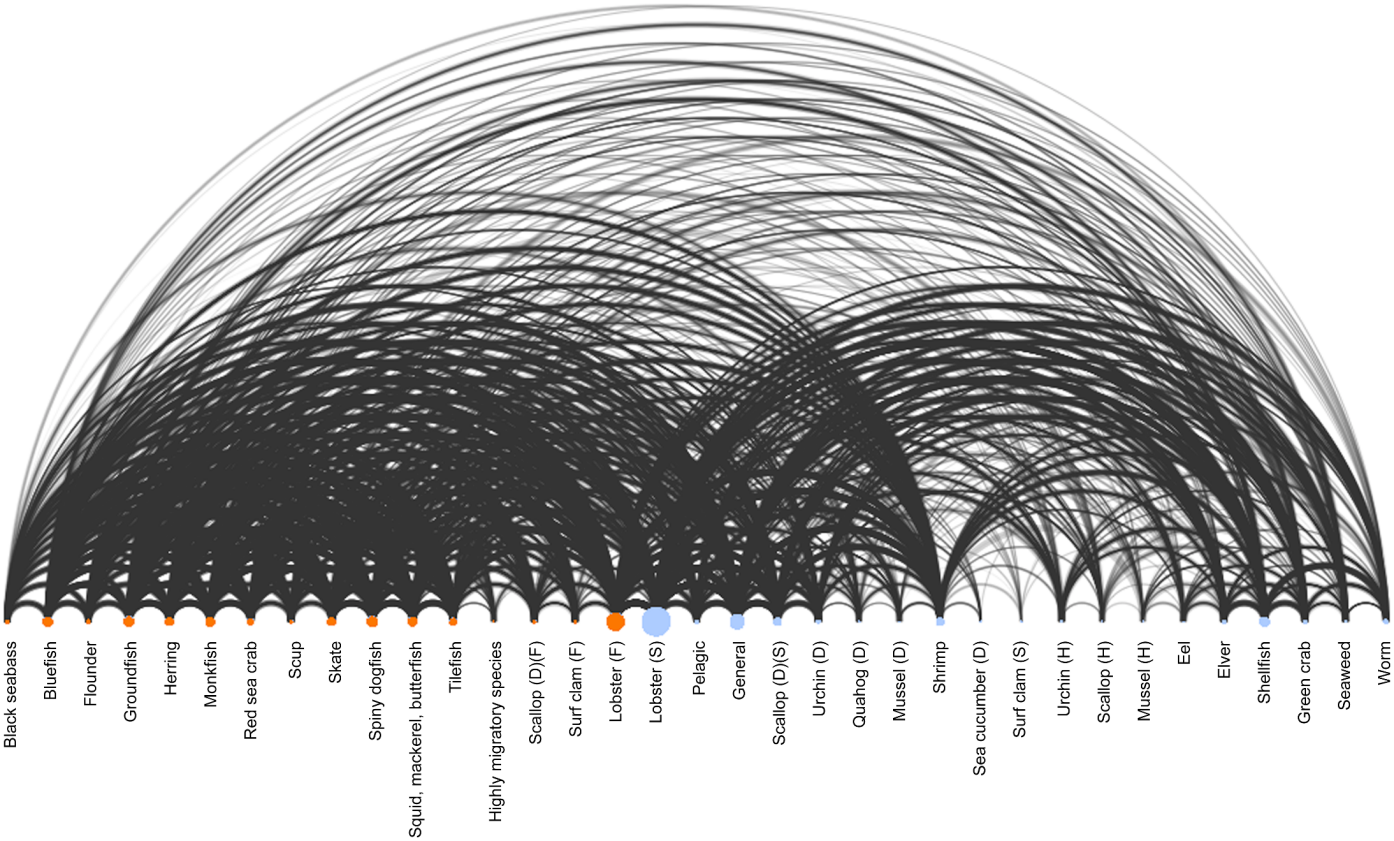
Fisher connectivity. Arc diagram depicting fishers’ (n = 8,576) connections to state and federal fisheries based on the portfolio of licenses that individuals hold. Fisheries are connected by arcs in instances where at least one fisher has access to both fisheries. Intensity and thickness of arcs are based on the number of links (i.e., number of harvesters). Node size reflects overall connectedness of each fishery. Note: (1) greater connectivity within federal fisheries (orange nodes); (2) high overall connectivity to the state and federal lobster fisheries; and (3) relatively weak links between state and federal fisheries. In cases where multiple license types exist for a single species: (D) = drag gear; (H) = hand harvesting; (S) = state license; (F) = federal license.

To identify the relationships that fishers in Maine have to different fisheries at the state and federal levels, we constructed a social-ecological network based on the different licensing assemblages that individuals hold, where the network edges describe licenses that are held by one or more fisher (Fig 1). Fishers are most highly connected to the state (degree centrality = 0.099) and federal (degree centrality = 0.093) lobster fisheries, which exhibit a degree centrality more than 70% higher than the third most central fishery. This result highlights the overall socioeconomic importance of the lobster fishery, which accounted for 81% of the overall ex-vessel value of fisheries in 2015 [25]. However, we also find that fishers have numerous and diverse connections to other fisheries that span gear types, jurisdictions, and geographies. These connections make the system appear more similar to the complex ecological system in the Gulf of Maine, with its myriad of biological connections [26], than the siloed regulations in place to govern the system [16].

### Types of fishers

**Fig 2:**
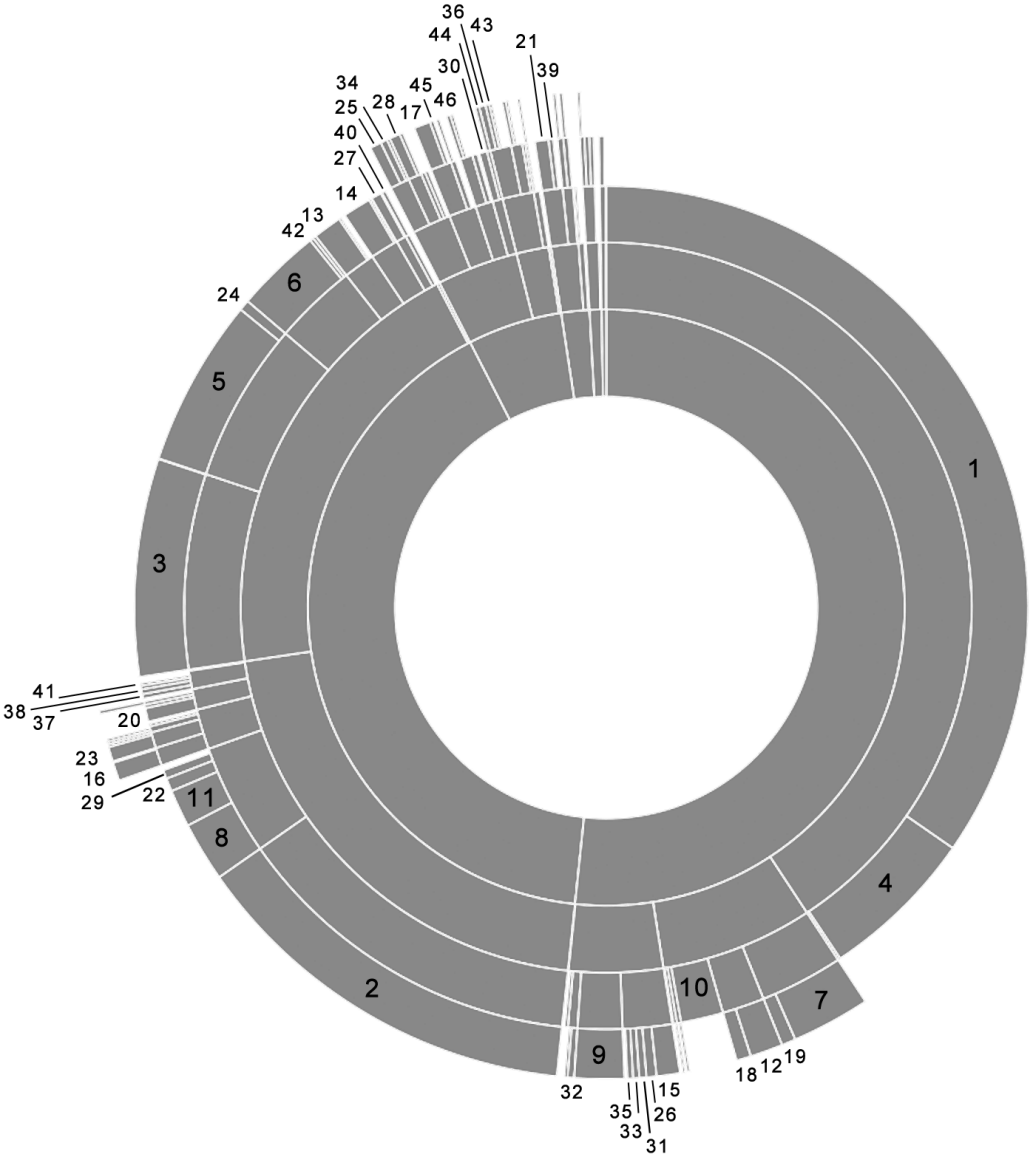
Typology. Illustration of the hierarchical typology of fisheries based on the assemblages of state and federal licenses commercial fishers in Maine held during 2014. Typology includes five levels starting with Level 1 in the center and moving outwards to Level 5. Each level (moving away from the center) is progressively more narrowly defined and therefore more homogenous. Numbers in the illustration correspond to the ID numbers in Table 2, which provides a description of each portfolio type with at least 10 individuals. The illustration does not include groups with fewer than 5 individuals although the results reported in this paper include data from all groups (n = 620).

We used a multivariate hierarchical cluster analysis to create a multi-level nested typology of the commercial fishing sector by classifying Maine fishers into portfolio types based on their individual assemblages of commercial fishing licenses. We employed this method as a novel way to simultaneously present a general (simplistic) and detailed (complex) description of the types of fishers in the system so as to be applicable to different types of research and policy analysis. This approach reveals the linkages between fisheries at relative scales of resolution. The first level of the typology provides a coarse separation of the fishing fleet with each subsequent level representing increasingly more distinct clusters until each cluster is unique (generally at the fourth or fifth level of the analysis). Our analysis revealed 620 unique types of fishers (S1 Table). Results in Fig 2 and Table 2 focus on the 46 types in the typology with at least 10 individuals, accounting for 88% of fishers in Maine, while the remaining results presented in this paper include all 620 groups. The heterogeneity identified in this analysis is commonly overlooked in descriptions of the fishing sector in Maine specifically, and in fisheries in general [27]. More commonly, fishers are described within the context of a particular fishery or simply as a largely monolithic group that is overly dependent on one species, in Maine the lobster fishery. Of the 620 groups, 66% of the fishers have one license.

**Table 2:** Primary types of fishers.

ID	License(s)	License(s) per individual	Number of individuals	Type
1	Lobster (State)	1	2832	State
2	Shellfish	1	1114	State
3	Marine Worm	1	609	State
4	Lobster (States), Lobster (Federal)	2	489	State
5	General Category	1	471	State
6	Elver	1	241	State
7	Lobster (State), General Category	2	220	State
8	Lobster (State), Shellfish	2	167	State
9	Lobster (Federal)	1	139	Federal
10	Lobster (State), Lobster (Federal), General Category	3	132	State/Federal
11	General Category, Shellfish	2	105	State
12	Lobster (State), Shrimp	2	91	State
13	Seaweed	1	80	State
14	Shellfish, Marine Worm	2	79	State
15	Lobster (State), Scallop (State) (Drag)	2	65	State
16	Green crab	1	47	State
17	Lobster (State), Lobster (Federal), General Category, Shrimp	4	42	State/Federal
18	Lobster (State), Lobster (Federal), Shrimp	3	41	State/Federal
19	Lobster (State), General Category, Shrimp	3	40	State
20	Elver, Lobster (State)	2	38	State
21	Urchin (Hand)	1	38	State
22	Elver, Shellfish	2	37	State
23	Green crab, Shellfish	2	37	State
24	General Category, Worm	2	30	State
25	Lobster (State), Lobster (Federal), General Category, Scallop (State) (Drag)	4	29	State/Federal
26	Scallop (State) (Drag)	1	28	State
27	Pelagic	1	27	State
28	Lobster (State), General Category, Scallop (State) (Drag)	3	27	State
29	Lobster (State), General Category, Shellfish	3	25	State
30	Lobster (State), Scallop (State) (Drag), Shrimp	3	21	State
31	Lobster (State), Lobster (Federal), Scallop (State) (Drag)	3	20	State/Federal
32	Shrimp	1	18	State
33	Lobster (State), Scallop (State) (Drag), Shellfish	3	17	State
34	Lobster (State), Lobster (Federal), General Category, Scallop (State) (Drag), Shrimp	5	17	State
35	Lobster (State), Lobster (Federal), Shellfish	3	16	State
36	Lobster (State), Scallop (State) (Drag), Urchin (Drag)	3	16	State
37	Lobster (State), Marine Worm	2	13	State
38	Lobster (State), Shellfish, Marine Worm	3	13	State
39	Lobster (State), Urchin (Hand)	2	13	State
40	Green crab, Worm	2	11	State
41	Green crab, Shellfish, Marine Worm	3	10	State
42	Elver, General Category	2	10	State
43	Lobster (State), General Category, Scallop (State) (Drag), Urchin (Drag)	4	10	State
44	Lobster (State), Lobster (Federal), Scallop (State) (Drag), Shrimp	4	10	State/Federal
45	Lobster (State), Lobster (Federal), General Category, Pelagic, Shrimp	5	10	State/Federal
46	Lobster (State), General Category, Pelagic	3	10	State

Description of the license portfolios of groups in the typology with 10 or more individuals in 2014 (n = 46). ID numbers correspond to the labels in Fig 2.

### Heterogeneity within the fishing fleet

**Fig 3:**
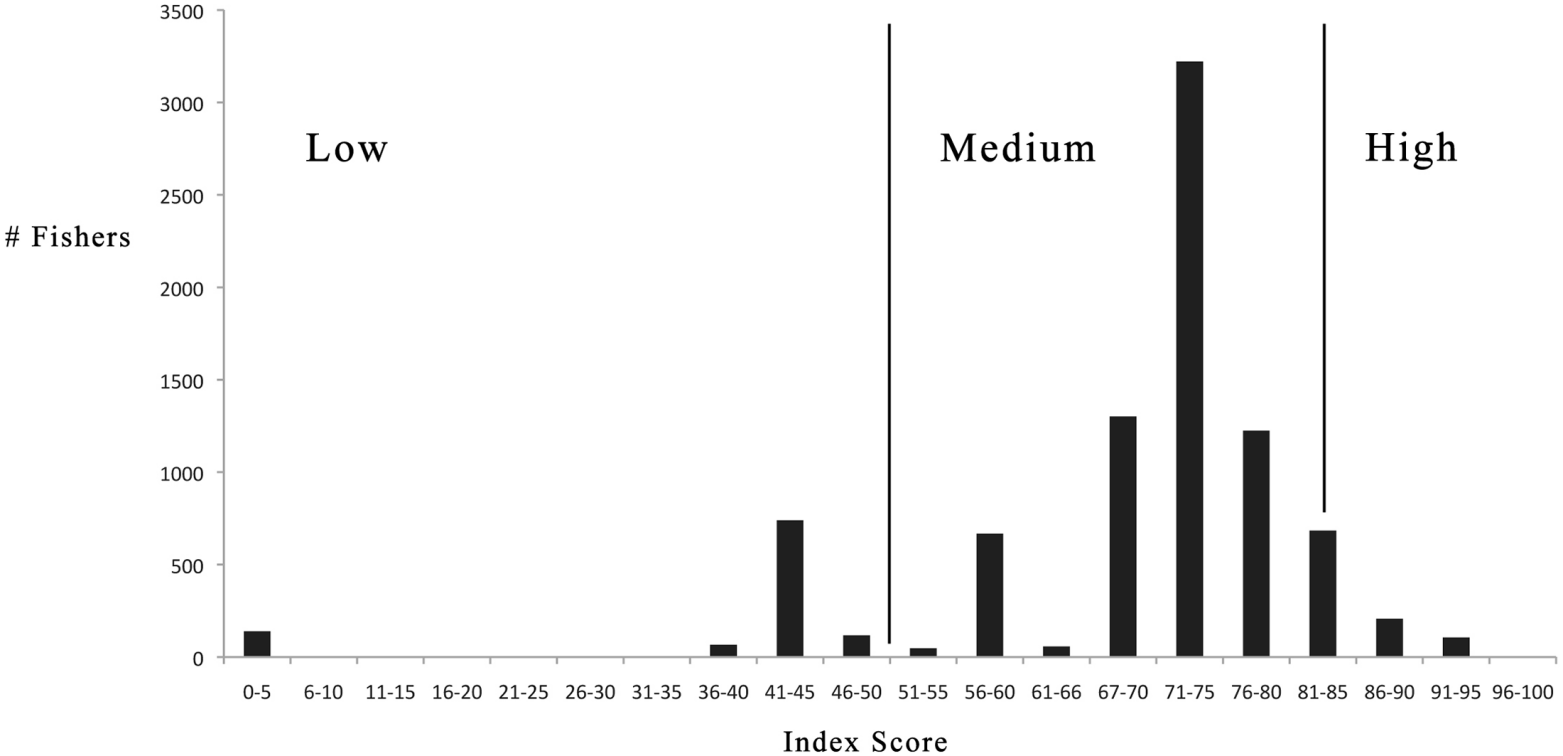
Adaptability index. Histogram depicting Adaptability Index scores (n = 620). Scores range from low (≤50) to medium (51–82) to high (≥83). Scores for each group in the typology are presented in S1 Table.

To understand how different combinations of licenses influence individuals’ adaptive capacity, we employed a modified Delphi approach to elicit criteria from fisheries experts in Maine. Briefly, this process involved interviewing a set of fisheries experts (n = 13) to elicit input on factors influencing fishers’ ability to adapt to change without leaving the commercial fishing sector altogether. Interviewees responded to several open-ended questions and then were invited to react to a set of factors that were identified beforehand through a literature review and pre-interviews. Interviews were used to identify a draft set of attributes that influence adaptive capacity and underlying criteria to evaluate them systematically. These were given back to experts and their feedback was used to refine and finalize the criteria. The criteria, outlined in detail in the methods section and supplemental materials (S2 Table), are (1) market stability, (2) economic value, (3) status of the stock, (4) governance structure, (5) geographic range, and (6) gear type. These were used to develop an adaptability index against which each of the 620 portfolio types were scored. To do this, fisheries in each of the portfolios were evaluated based on attributes 1–4 and then each portfolio (as a whole) was assessed based on attributes 5 and 6. Through this assessment, values from 1 (low) to 3 (high) were assigned to each attribute based on the criteria described in the methods. In several instances values of 0 were also given when a regulation negated the relevance of other attributes. Scores ranging from 0 to 50, 51 to 82, and 83 to 100 represent low, medium, and high index scores. Scores for portfolio types range from 0 to 94 (Fig 3). The average unweighted score for the 620 groups in the typology is 68 out of a possible 100 and the median is 72. For index scores for each group, see S1 Table. Mean adaptability scores are generally lower among fishers holding state licenses (67) than those also (or only) holding federal licenses (72). The highest scoring fishers hold multiple fishing licenses and participate in both state and federal fisheries. The composition of portfolio types with low index scores is more diverse. Extreme cases include individuals holding a single license for a low value fishery (e.g., general category (n = 47)) or one that is closed (e.g., shrimp, n = 18)). In another instance, 139 individuals hold a federal lobster license, but because they do not also have a state lobster license, it cannot be used in Maine, reducing their potential adaptive capacity. There are also instances where individuals hold a license for a species that is not readily available in Maine, such as bluefish (n = 1). Overall these scores reflect both the different levels of adaptive capacity across the fleet and also illustrate the large number of fishers who have licensing portfolios that do not facilitate adaptation.

## Discussion

Fisheries are largely managed on a species by species basis, as is corresponding research about their human dimensions. Inattention to the diverse connections that fishers have to fisheries, however, obscures meaningful differences that influence how individuals interact with the marine environment and respond to change. In this paper, we bring explicit attention to the heterogeneity of fishing strategies employed in Maine and evaluate the adaptive capacity afforded by each, finding that fishers’ ability to respond to change is highly variable due to differences in the stability of markets, value of species, status of stocks, and underlying governance structure. Understanding cross-fishery connectivity is critical to devising effective management strategies that support fishers and healthy fisheries in a changing seascape worldwide.

Each combination affords fishers access to a different set of opportunities that contributes to different levels of adaptive capacity. Of the groups in the typology that include lobster licenses, for example, the mean adaptability scores ranged from 0 to 94. Such variability is seldom acknowledged, but has important implications in terms of how individuals will (or can) respond to change. The differences in adaptive capacity correspond to differences in market stability, economic value, and stock status of individual fisheries as well as the geographic and gear diversity that fishers maintain across fisheries. For example, 167 (1.9%) fishers hold a state lobster license and a shellfish license and 38 (0.4%) hold a state lobster license and an elver license exclusively. In both instances, these fishers have access to two relatively valuable species. These fishers also benefit from being diversified in terms of geography and gear because the lobster fishery occurs in the ocean and requires a boat and traps, whereas the other two fisheries take place on the coast and in rivers and require a rake and dip net or fyke net (which are fairly inexpensive to construct or obtain). Fishers participating in these fisheries also gain access to social capital derived from local management and fishing associations, such as the Maine Elver Association, that have been created to support these fisheries. This stands in contrast to less commercially valuable fisheries and those that are not well organized, such as the marine worm fishery (n = 609 (7.1%)), which has had a stable market, but is not managed at a local level and is not organized. Such differences have a direct impact on the range of possibilities available to fishers when negotiating various challenges, whereby affecting their adaptive capacity. Delimiting these differences brings explicit attention to the multiplicity of connections that fishers have to different fisheries and begins to make apparent that these differences, which cannot be seen by taking a narrow single-species approach to studying fisheries, have real implications for if and how industry members interact with marine resources and adapt to future change.

The results presented in this paper serve to highlight the uneven capacity of the fishing fleet in Maine to adapt based on the criteria used in our assessment. To a certain extent, these data give reason for anxiety about the future of the commercial fishing sector. Based on our evaluation of the license typology, 88% of fishers (n = 7,550) have low (≤50) to medium (≤82) adaptive capacity given their existing licensing portfolios. However, it is important that these results are understood within the broader context in which many commercial fishers operate. Fishers’ ability to adapt to change is dependent upon a multitude of factors of which access to licenses is only one. These additional factors include but are not limited to opportunities for employment outside of the fishing sector, individual knowledge and expertise, community connections, personal and family finances, and general open-mindedness. Further research is needed to more fully understand how these factors influence adaptability, as it is likely that they also play an important role. In some instances, these factors will likely further reduce individuals’ ability to anticipate and respond to changes, while in other cases they will increase individuals’ capacity, whereby mitigating some of the negative implications of our results based solely on license portfolios.

It is also important to acknowledge that fishers are often extremely adept at problem solving. Therefore, we should not necessarily assume that having moderate or even low adaptive capacity will necessarily prevent adaptation altogether. An alternative hypothesis is that it could foster innovation and catalyze fishers’ willingness to adapt. This hypothesis is based on the logic that change is often driven by necessity and it is at these moments of uncertainty, increased vulnerability, and elevated risk perception when individuals are compelled to take risks, experiment, and pursue new ways of doing business (even if they are not well positioned to try) [28]. If this alternate scenario is correct, then the current state of the fishing sector in Maine could present the conditions necessary for experimentation and risk taking. In practical terms, this may mean the formation of new alliances between sectors of the fishing community and external stakeholders, adoption of different marketing strategies, willingness to try new governance structures, or testing of alternative ways to making a living off the water. Signs of these types of innovation may already be starting to become visible. In recent years, there have been a wave of fishers experimenting with direct marketing arrangements [29], there is an increasing number of people entering the aquaculture sector, and fisheries managers at the state level are working with fishers to implement new management regimes and utilize new technologies to improve monitoring and accountability. Fisheries managers and policymakers will need to play a critical role in facilitating and ultimately institutionalizing innovation and therefore should be mindful that those who may be most willing to experiment and innovate may also have the least capacity. This will likely include individuals who are poorly diversified in terms of geography and gear and/or dependent on fisheries that are low value or have unstable stocks and markets. It will also include fishers that do not have a mechanism to participate in the management process formally or lack industry groups that actively advocate for their needs. Accordingly, these individuals are apt not to be the “usual suspects” who have existing relationships with managers or policymakers. Therefore managers and policymakers will need to find creative ways to identify, listen to, and engage with these fishers to better understand the complex needs of the industry.

Ultimately, while the exact responses that fishers will have to future changes remain unknown, more explicit attention to the diverse and complex connections fishers have to fisheries is vital to understanding the vulnerability of the fishing industry. In this paper, we focus on fisheries in Maine, yet complex fisher-fisheries connections are ubiquitous in fisheries in the United States and more broadly. Understanding these linkages will contribute to our collective understanding of fisher behavior and social-ecological dynamics in marine systems.

## Supporting information

S1 Table Adaptability index. List of license groups in the typology along with their Adaptability Index Scores.
(DOCX)

S2 Table Adaptive capacity evaluation. Evaluation of adaptive capacity of fishing portfolios based on the attributes identified by fisheries experts in Maine.
(DOCX)
