# Supplementary material for: Uneven adaptive capacity among fishers in a sea of change
Source: PLoS One. 2017 Jun 12;12(6):e0178266. doi: 10.1371/journal.pone.0178266 (PMC5467827; doi:10.1371/journal.pone.0178266)
Supplement: S1 Table — List of license groups in the typology along with their Adaptability Index Scores. (DOCX) [file pone.0178266.s001.docx]

| **S1 Table. Adaptability Index** |  |  |
| --- | --- | --- |
| List of license groups in the typology along with their Adaptability Index Scores. |  |  |
|  |  |  |
| **License Key** | **Code** |  |
| Pelagic and Anadromous | Pel |  |
| Mussel (Hand) | MuH |  |
| Mussel (Drag) | MuD |  |
| Spiny Dogfish | SPI |  |
| Eel | Eel |  |
| Elver | Elv |  |
| Surf (State) | SurS |  |
| Surf (Federal) | SurF |  |
| Herring | HER |  |
| Lobster (State) | LobS |  |
| Lobster (Federal) | LobF |  |
| Red Sea Crab | RSC |  |
| Green Crab | Gre |  |
| Quahog (Drag) | QuD |  |
| Monkfish | MON |  |
| General | Gen |  |
| Worm | Wor |  |
| Sea Cucumber | Sea |  |
| Scallop (State) (Drag) | ScSD |  |
| Scallop (Federal) (Drag) | ScFD |  |
| Scallop (Hand) | ScH |  |
| Seaweed | Wee |  |
| Shrimp | Shr |  |
| Skate | SKA |  |
| Shellfish | She |  |
| Urchin (Drag) | UrD |  |
| Urchin (Hand) | UrH |  |
| Groundfish | GRO |  |
| Flounder | FLO |  |
| Highly Migratory Species | HMS |  |
| Black Sea Bass | BSB |  |
| Bluefish | BLU |  |
| Scup | SCU |  |
| Squid, Mackerel, Butterfish | SMB |  |
| Tilefish | TIL |  |
|  |  |  |
| **Group** | **Index Score** | **Number** |
| LobS | 72 | 2832 |
| She | 67 | 1114 |
| Wor | 56 | 609 |
| LobSLobF | 78 | 489 |
| Gen | 42 | 471 |
| Elver | 44 | 241 |
| LobSGen | 72 | 220 |
| LobSShe | 83 | 167 |
| LobF | 0 | 139 |
| LobSLobFGen | 78 | 132 |
| GenShe | 78 | 105 |
| LobSShr | 78 | 91 |
| Wee | 50 | 80 |
| SheWor | 67 | 79 |
| LobSScSD | 78 | 65 |
| Gre | 39 | 47 |
| LobSLobFGenShr | 83 | 42 |
| LobSLobFShr | 83 | 41 |
| LobSGenShr | 78 | 40 |
| UrH | 61 | 38 |
| ElverLobS | 83 | 38 |
| ElverShe | 67 | 37 |
| GreShe | 78 | 37 |
| GenWor | 67 | 30 |
| LobSLobFGenScSD | 83 | 29 |
| ScSD | 72 | 28 |
| Pel | 51 | 27 |
| LobSGenScSD | 78 | 27 |
| LobSGenShe | 83 | 25 |
| LobSScSDShr | 78 | 21 |
| LobSLobFScSD | 83 | 20 |
| Shr | 33 | 18 |
| LobSLobFGenScSDShr | 83 | 17 |
| LobSScSDShe | 89 | 17 |
| LobSScSDUrD | 78 | 16 |
| LobSLobFShe | 89 | 16 |
| LobSUrH | 78 | 13 |
| LobSWor | 83 | 13 |
| LobSSheWor | 83 | 13 |
| GreWor | 67 | 11 |
| ElverGen | 56 | 10 |
| GreSheWor | 78 | 10 |
| LobSGenScSDUrD | 78 | 10 |
| LobSLobFScSDShr | 83 | 10 |
| LobSGenPel | 83 | 10 |
| LobSLobFGenPelShr | 94 | 10 |
| ElverWor | 56 | 9 |
| ScHUrH | 72 | 9 |
| LobSGenScSDShr | 78 | 9 |
| ElverLobSShe | 83 | 9 |
| LobSSheShr | 89 | 9 |
| LobSGenScSDShe | 89 | 9 |
| GenShr | 47 | 8 |
| GenPel | 53 | 8 |
| ScH | 72 | 8 |
| SheUrH | 72 | 8 |
| GreLobSLobFGen | 78 | 8 |
| LobSPel | 83 | 8 |
| LobSLobFGenScSDUrD | 83 | 8 |
| GreGen | 42 | 7 |
| Qua | 56 | 7 |
| GenWee | 61 | 7 |
| GreLobSGen | 72 | 7 |
| LobFBLUMONGOUNDHERRSCSKADOGSMBTILE | 72 | 7 |
| GreGenShe | 78 | 7 |
| GenSheWor | 78 | 7 |
| ElverLobSGen | 83 | 7 |
| LobSScSDSheUrD | 89 | 7 |
| LobSGenScSDSheUrD | 89 | 7 |
| Eel | 44 | 6 |
| BLUDOG | 50 | 6 |
| ElverSheWor | 67 | 6 |
| ScSDUrD | 72 | 6 |
| LobSScH | 78 | 6 |
| GenScSD | 78 | 6 |
| ScSDShe | 83 | 6 |
| LobSLobFGOUND | 83 | 6 |
| LobSLobFGenShe | 89 | 6 |
| LobSGenPelScSDShr | 89 | 6 |
| LobSLobFGenScSDShe | 94 | 6 |
| LobFGen | 42 | 5 |
| MuH | 56 | 5 |
| GOUND | 49 | 5 |
| BSBBLUFLOMONGOUNDHERRSCSCUSKADOGSMBTILE | 72 | 5 |
| LobFBSBBLUFLOMONGOUNDHERRSCSCUSKADOGSMBTILE | 72 | 5 |
| LobSScHUrH | 78 | 5 |
| LobFSurFScFDBLUMONGOUNDHERRSCSKADOGSMBTILE | 72 | 5 |
| GreLobSShe | 83 | 5 |
| LobSLobFScSDUrD | 83 | 5 |
| GreLobSLobFGenShr | 83 | 5 |
| ElverLobSScSD | 89 | 5 |
| LobSGenPelScSD | 89 | 5 |
| LobSLobFGenPel | 89 | 5 |
| GenPelScSDShr | 89 | 5 |
| LobSLobFGenScSDSheUrD | 94 | 5 |
| EelGre | 44 | 4 |
| WeeWor | 56 | 4 |
| BLUFLODOG | 50 | 4 |
| UrD | 61 | 4 |
| ScHSheUrH | 78 | 4 |
| ScSDShr | 72 | 4 |
| BLUMONGOUNDHERRSCSKADOGSMBTILE | 72 | 4 |
| LobFSurFBLUMONGOUNDHERRSCSKADOGSMBTILE | 72 | 4 |
| LobFBLUMONGOUNDHERDOGSMB | 72 | 4 |
| ElverGenShe | 78 | 4 |
| GenWeeShe | 78 | 4 |
| LobSGenUrD | 78 | 4 |
| LobSUrD | 78 | 4 |
| GenBLUMONGOUNDHERSKADOGSMBTILE | 78 | 4 |
| LobSSheUrH | 83 | 4 |
| LobSLobFGenScSDShrUrD | 83 | 4 |
| ElverLobSLobFGen | 89 | 4 |
| GenScSDShe | 89 | 4 |
| LobSGenWeeShr | 89 | 4 |
| LobSLobFGenPelScSDShr | 94 | 4 |
| GreLobS | 72 | 3 |
| ElverMuHShe | 72 | 3 |
| ScHShe | 78 | 3 |
| BSBBLUFLOMONGOUNDHERSCUSKADOGSMBTILE | 72 | 3 |
| ElverGreShe | 78 | 3 |
| LobSGenUrH | 78 | 3 |
| GreLobSGenScSD | 78 | 3 |
| GreLobSGenShr | 78 | 3 |
| GenScSDShr | 78 | 3 |
| ElverLobSGenShe | 83 | 3 |
| ElverGreLobSGen | 83 | 3 |
| LobSGenSheWor | 83 | 3 |
| ElverScHUrH | 89 | 3 |
| LobSLobFUrH | 83 | 3 |
| ScSDSheUrD | 83 | 3 |
| LobSLobFGenMuDScSD | 83 | 3 |
| LobSLobFPel | 89 | 3 |
| LobSGenSheShr | 89 | 3 |
| LobSGenPelShr | 89 | 3 |
| LobSLobFSheShr | 94 | 3 |
| LobSLobFScSDShe | 94 | 3 |
| ElverLobSLobFGenScSD | 94 | 3 |
| LobSLobFGenPelScSDShrUrD | 94 | 3 |
| LobSLobFGenSheShr | 94 | 3 |
| ElverWee | 50 | 2 |
| SCD | 44 | 2 |
| MuD | 56 | 2 |
| GreGenPel | 53 | 2 |
| LobFGOUND | 49 | 2 |
| ElverPel | 56 | 2 |
| WeeUrH | 67 | 2 |
| ElverUrH | 67 | 2 |
| GenMuD | 61 | 2 |
| ElverGenPel | 56 | 2 |
| BSBBLUFLODOG | 50 | 2 |
| GreGenWee | 61 | 2 |
| UrHWor | 67 | 2 |
| WeeShe | 67 | 2 |
| WeeSheWor | 67 | 2 |
| LobFGOUNDHER | 67 | 2 |
| MuHShe | 72 | 2 |
| GenScHUrH | 78 | 2 |
| GenScH | 78 | 2 |
| MuDScSD | 72 | 2 |
| LobFBLUMONGOUNDHERRSCSKADOGSMB | 72 | 2 |
| LobFBLUMONGOUNDHERSKADOGSMB | 72 | 2 |
| BLUMONGOUNDHERSKADOGSMBTILE | 72 | 2 |
| EelShe | 78 | 2 |
| ElverGreSheWor | 78 | 2 |
| GreGenSheWor | 78 | 2 |
| ElverGenSheWor | 78 | 2 |
| PelShe | 78 | 2 |
| GreLobSLobF | 78 | 2 |
| GenSheUrH | 78 | 2 |
| ScSDScH | 78 | 2 |
| LobSScSDShrUrD | 78 | 2 |
| LobSScSDSCD | 78 | 2 |
| LobSGenScSDShrUrD | 78 | 2 |
| LobFScFDBLUMONGOUNDHERRSCSKADOGSMBTILE | 72 | 2 |
| LobFScFDBSBBLUFLOMONGOUNDHERRSCSCUSKADOGSMBTILE | 72 | 2 |
| LobFSurFScFDBSBBLUFLOMONGOUNDHERRSCSCUSKADOGSMBTILE | 72 | 2 |
| ElverGreLobSGenShe | 83 | 2 |
| LobSWeeShe | 83 | 2 |
| LobSGenMuHShe | 83 | 2 |
| LobSGenWor | 83 | 2 |
| ElverLobSWor | 83 | 2 |
| GreLobSSheWor | 83 | 2 |
| GreLobSWor | 83 | 2 |
| LobSLobFGenGOUND | 83 | 2 |
| ElverLobSSheUrH | 83 | 2 |
| LobSScSDScHUrH | 83 | 2 |
| GenSheUrD | 83 | 2 |
| LobSLobFGenQuaScSDShr | 83 | 2 |
| LobSLobFGenShrGOUND | 83 | 2 |
| LobSGenPelShe | 83 | 2 |
| ElverLobSGenPel | 83 | 2 |
| GreLobSGenWee | 83 | 2 |
| LobSLobFBLUMONGOUNDHERRSCSKADOGSMBTILE | 83 | 2 |
| LobSLobFBLUMONGOUNDHERSKADOGSMB | 83 | 2 |
| LobSLobFBLUMONGOUNDSKADOGSMB | 83 | 2 |
| LobSLobFBLUMONGOUNDHERSKADOGSMBTILE | 83 | 2 |
| LobSLobFShrBLUMONGOUNDHERRSCSKADOGSMBTILE | 83 | 2 |
| LobSLobFSheWor | 89 | 2 |
| LobSScSDSheShr | 89 | 2 |
| LobSGenScSDSheShr | 89 | 2 |
| LobSGenPelScSDShe | 89 | 2 |
| ElverLobSGenPelScSDShr | 89 | 2 |
| ElverLobSGenShr | 89 | 2 |
| GreLobSGenPelWeeShr | 89 | 2 |
| LobSLobFGenScSDSheShr | 94 | 2 |
| LobSLobFGenPelScSDShrBLUMONGOUNDHERRSCSKADOGSMBTILE | 94 | 2 |
| BLU | 39 | 1 |
| EelGen | 44 | 1 |
| LobFBLURSC | 44 | 1 |
| EelGreGen | 44 | 1 |
| GreGenShr | 47 | 1 |
| LobFBLUDOG | 50 | 1 |
| LobFSKADOG | 50 | 1 |
| EelGenShr | 50 | 1 |
| GrePel | 51 | 1 |
| GOUNDSKASMB | 49 | 1 |
| LobFMONGOUND | 50 | 1 |
| BLUFLOMONGOUNDSMB | 50 | 1 |
| BLUMONGOUNDSKA | 50 | 1 |
| ElverGre | 56 | 1 |
| ElverEelGen | 56 | 1 |
| ElverEel | 56 | 1 |
| LobFWor | 56 | 1 |
| ElverWeeUrH | 67 | 1 |
| GenQua | 61 | 1 |
| SurFMONGOUNDSKASMB | 56 | 1 |
| GenPelShr | 58 | 1 |
| GOUNDDOGSMB | 54 | 1 |
| BSBGOUNDDOGSMB | 54 | 1 |
| GOUNDSCUSMBTILE | 54 | 1 |
| LobFBLUMONGOUNDSKADOGSMBTILE | 56 | 1 |
| MONGOUNDRSCSKADOGSMBTILE | 56 | 1 |
| BLUFLOGOUNDDOGSMB | 54 | 1 |
| BLUGOUNDDOGSMBTILE | 54 | 1 |
| LobFBLUMONGOUNDDOGSMB | 56 | 1 |
| BLUGOUNDSKADOGTILE | 54 | 1 |
| MONGOUNDSKADOGSMB | 56 | 1 |
| BLUFLOMONGOUNDRSCSCUSKADOGSMBTILE | 56 | 1 |
| BSBBLUFLOMONGOUNDRSCSCUSKADOGSMBTILE | 56 | 1 |
| BSBBLUFLOMONGOUNDSCUDOGSMB | 56 | 1 |
| BSBBLUFLOMONGOUNDSCUDOGSMB | 56 | 1 |
| BLUFLOGOUNDSCUDOGSMB | 54 | 1 |
| BSBBLUFLOGOUNDSCUSMBTILE | 54 | 1 |
| BSBBLUFLOGOUND | 54 | 1 |
| WeeUrHWor | 67 | 1 |
| ElverShrUrH | 72 | 1 |
| ElverEelUrH | 72 | 1 |
| GenShrUrH | 72 | 1 |
| EelGreGenWee | 61 | 1 |
| SurFBLUDOGSMB | 61 | 1 |
| GenGOUND | 60 | 1 |
| GenBLUGOUNDSMB | 60 | 1 |
| ShrFLOMONGOUNDSCUSKADOGSMB | 61 | 1 |
| GenBSBBLUFLOGOUNDSCUSMBTILE | 60 | 1 |
| ElverWeeShe | 67 | 1 |
| LobFShe | 67 | 1 |
| GenWeeWor | 67 | 1 |
| GreGenMuDWee | 72 | 1 |
| EelGreGenPelWor | 67 | 1 |
| GenMuDPelQuaShr | 72 | 1 |
| BLUMONGOUNDHERSMB | 67 | 1 |
| ScFD | 67 | 1 |
| LobFGOUNDHERDOG | 72 | 1 |
| LobFDOG | 72 | 1 |
| LobFScSD | 72 | 1 |
| LobFScFD | 67 | 1 |
| LobFMONGOUNDHERDOG | 72 | 1 |
| SheUrHWor | 72 | 1 |
| ScHUrHWor | 78 | 1 |
| GreScHUrH | 78 | 1 |
| ScHWee | 78 | 1 |
| QuaScSD | 72 | 1 |
| MuDQuaScSD | 72 | 1 |
| ScSDSCDShr | 72 | 1 |
| LobFMONGOUNDHERRSCSKADOGSMB | 72 | 1 |
| BLUMONGOUNDHERRSCSCUSKADOGSMBTILE | 72 | 1 |
| BLUGOUNDHERDOGSMB | 72 | 1 |
| BLUMONGOUNDHERDOGSMB | 72 | 1 |
| LobFBLUFLOMONGOUNDHERSKADOGSMB | 72 | 1 |
| LobFBLUGOUNDHERDOGSMB | 72 | 1 |
| LobFGOUNDHERSKADOGSMB | 72 | 1 |
| LobFMONGOUNDHERDOGSMB | 72 | 1 |
| MONGOUNDHERSKASMBTILE | 72 | 1 |
| ScFDMONGOUNDHERSKA | 67 | 1 |
| LobFSurFScFDBLUMONGOUNDHERSMB | 67 | 1 |
| SurFScFDBLUGOUND | 67 | 1 |
| LobFSurFMONGOUNDHERSCUDOGSMB | 72 | 1 |
| LobFSurFBSBBLUFLOMONGOUNDHERHMSRSCSCUSKADOGSMBTILE | 72 | 1 |
| BSBBLUFLOMONGOUNDHERSCUSKADOGSMB | 72 | 1 |
| LobFBLUFLOMONGOUNDHERRSCSCUSKADOGSMBTILE | 72 | 1 |
| LobFBSBBLUMONGOUNDHERSCUSKADOGSMBTILE | 72 | 1 |
| BSBBLUFLOGOUNDHERSCUSKASMBTILE | 72 | 1 |
| BSBBLUFLOGOUNDHERSCUDOGSMB | 72 | 1 |
| SheUrD | 78 | 1 |
| SheShr | 78 | 1 |
| ElverGreGenShe | 78 | 1 |
| EelGenShe | 78 | 1 |
| GreGenWeeShe | 78 | 1 |
| GreMuHShe | 78 | 1 |
| ElverGreGenSheWor | 78 | 1 |
| GreLobFSheWor | 78 | 1 |
| GreWeeSheWor | 78 | 1 |
| GenMONGOUNDHERSMB | 78 | 1 |
| ScFDMONDOG | 72 | 1 |
| LobSLobFGenDOG | 78 | 1 |
| GenSheUrHWor | 78 | 1 |
| ScSDUrH | 78 | 1 |
| ElverGreScHSheUrH | 83 | 1 |
| ElverGenScHSheUrH | 83 | 1 |
| ElverGenSheUrH | 78 | 1 |
| GenScHSheUrH | 83 | 1 |
| LobSGenScH | 78 | 1 |
| ElverEelGreGenMuHSheUrH | 78 | 1 |
| EelMuHScHShe | 83 | 1 |
| LobSQua | 78 | 1 |
| LobSSurS | 78 | 1 |
| LobSGenMuDQuaScSD | 78 | 1 |
| LobSMuDScSD | 78 | 1 |
| GreGenScSDSCDShrUrD | 78 | 1 |
| LobSGenQuaScSDUrD | 78 | 1 |
| LobSShrUrD | 78 | 1 |
| GenScSDUrD | 78 | 1 |
| LobSGenMuDScSDShr | 78 | 1 |
| LobSGenShrUrD | 78 | 1 |
| LobSSurSShr | 78 | 1 |
| GreLobSGenScSDShrUrD | 78 | 1 |
| GreLobSGenScSDShr | 78 | 1 |
| EelGreLobSGenShr | 78 | 1 |
| EelGreGenWeeShe | 78 | 1 |
| EelLobSGenScSD | 78 | 1 |
| GreGenPelShe | 78 | 1 |
| GenPelWeeSheWor | 78 | 1 |
| GenShrMONGOUNDHERSMB | 78 | 1 |
| LobFBLUMONGOUNDHERRSCDOGSMB | 78 | 1 |
| LobFScFDMONGOUNDSKADOGSMBTILE | 72 | 1 |
| LobFBLUFLOMONGOUNDHERRSCSKADOGSMBTILE | 78 | 1 |
| LobFBLUFLOMONGOUNDHERSKADOGSMBTILE | 78 | 1 |
| LobFScFDBLUFLOMONGOUNDHERRSCSKADOGSMBTILE | 78 | 1 |
| LobFBLUMONGOUNDHERRSCSKADOGTILE | 78 | 1 |
| ScFDBLUMONGOUNDHERRSCSKADOGSMBTILE | 72 | 1 |
| ScFDBLUMONGOUNDHERRSCSKADOGTILE | 72 | 1 |
| ScFDBLUMONHERRSCSKADOGSMBTILE | 72 | 1 |
| LobFScFDBLUMONGOUNDRSCSKADOGSMBTILE | 78 | 1 |
| LobFSurFBLUMONGOUNDHERRSCSKADOGSMB | 78 | 1 |
| LobFSurFScFDBLUMONGOUNDHERDOGSMBTILE | 78 | 1 |
| LobFSurFScFDBLUMONGOUNDRSCSKADOGSMBTILE | 78 | 1 |
| LobFSurFScFDBLUMONRSCSKADOGSMBTILE | 78 | 1 |
| LobFBLUMONHERRSCSKASMBTILE | 78 | 1 |
| GenBLUMONGOUNDHERRSCSKADOGSMBTILE | 78 | 1 |
| LobFSurFScFDBSBBLUFLOMONGOUNDHERRSCSKADOGSMBTILE | 72 | 1 |
| LobFScFDBLUMONDOGSMB | 72 | 1 |
| LobFScFDMONDOG | 72 | 1 |
| LobFScFDMONGOUNDHERSCUSKADOGSMB | 72 | 1 |
| LobFScFDFLOMONGOUNDDOG | 72 | 1 |
| LobFScFDFLOMONGOUNDHERRSC | 72 | 1 |
| LobFScFDBSBBLUFLOMONGOUNDHERRSCSCUSKADOGSMB | 72 | 1 |
| GenBSBBLUFLOMONGOUNDHERRSCSCUSKADOGSMBTILE | 78 | 1 |
| LobFGenBSBBLUFLOMONGOUNDHERRSCSCUSKADOGSMBTILE | 78 | 1 |
| GenSheShr | 83 | 1 |
| ElverGenSheShr | 83 | 1 |
| GreSheShr | 83 | 1 |
| GreLobSGenShe | 83 | 1 |
| ElverEelGreLobSShe | 83 | 1 |
| EelLobSGenWor | 83 | 1 |
| LobSWee | 83 | 1 |
| LobSGenWee | 83 | 1 |
| ElverEelLobS | 83 | 1 |
| ElverGreLobS | 83 | 1 |
| ScSDWor | 83 | 1 |
| GreLobSGenWor | 83 | 1 |
| ElverLobSGenSheWor | 83 | 1 |
| ElverLobSGenWor | 83 | 1 |
| ElverLobSSheWor | 83 | 1 |
| ElverGreLobSSheWor | 83 | 1 |
| ElverLobSPel | 83 | 1 |
| LobSLobFGOUNDDOG | 83 | 1 |
| LobSLobFShrDOG | 83 | 1 |
| LobSLobFMONGOUND | 83 | 1 |
| LobSLobFGOUNDHERSMB | 83 | 1 |
| LobSLobFGOUNDSMB | 83 | 1 |
| LobSLobFShrBLUMONHERSMB | 83 | 1 |
| LobSLobFBLUGOUNDHERSMB | 83 | 1 |
| LobSLobFGenBLUGOUNDDOG | 83 | 1 |
| LobSLobFBLUGOUNDHERDOG | 83 | 1 |
| LobSLobFBLUGOUND | 83 | 1 |
| LobSLobFShrBLUGOUNDHER | 83 | 1 |
| LobSLobFBLUHERSKA | 83 | 1 |
| LobSWeeSheUrHWor | 83 | 1 |
| ElverLobSUrHWor | 83 | 1 |
| ElverScHUrHWor | 89 | 1 |
| ScSDUrHWor | 83 | 1 |
| LobSWeeUrH | 83 | 1 |
| LobSGenScHWeeUrHWor | 83 | 1 |
| LobSGenWeeUrH | 83 | 1 |
| LobSScHWeeUrH | 83 | 1 |
| ElverScHSheUrH | 89 | 1 |
| ElverScH | 89 | 1 |
| LobSScSDScH | 83 | 1 |
| LobSGenScHShr | 83 | 1 |
| LobSScHShr | 83 | 1 |
| LobSScHShrUrH | 83 | 1 |
| ElverLobSGenScHUrH | 83 | 1 |
| LobSLobFGenScHUrH | 83 | 1 |
| LobSLobFScHUrH | 83 | 1 |
| LobSGenScSDScHUrH | 83 | 1 |
| GreLobSGenScHSheUrHWor | 83 | 1 |
| LobSLobFMuDScSD | 83 | 1 |
| LobSLobFQua | 83 | 1 |
| LobSLobFQuaScSD | 83 | 1 |
| MuDScSDWor | 83 | 1 |
| LobSLobFGenQuaScSDScFDUrD | 83 | 1 |
| LobSLobFMuDScSDUrD | 83 | 1 |
| MuDScSDSheUrD | 83 | 1 |
| LobSLobFQuaScSDUrD | 83 | 1 |
| LobSLobFUrD | 83 | 1 |
| LobSLobFScSDSCD | 83 | 1 |
| LobSLobFGenMuDScSDShrUrD | 83 | 1 |
| LobSLobFGenScSDSCD | 83 | 1 |
| LobSLobFGenScSDHERSKA | 83 | 1 |
| LobSLobFGenUrD | 83 | 1 |
| LobSLobFGenScSDGOUNDHER | 83 | 1 |
| LobSLobFGenScSDShrGOUND | 83 | 1 |
| LobSLobFGenScSDShrUrDGOUND | 83 | 1 |
| LobSLobFGenScSDUrDGOUND | 83 | 1 |
| LobSLobFScSDShrUrD | 83 | 1 |
| LobSLobFGenShrUrD | 83 | 1 |
| GreLobSLobFGenScSDShr | 83 | 1 |
| ElverLobSGenPelShe | 83 | 1 |
| LobSGenPelWee | 83 | 1 |
| EelLobSGenShe | 83 | 1 |
| ElverEelGreLobSGen | 83 | 1 |
| ElverEelGreLobSGenShe | 83 | 1 |
| ElverLobSGenPelWor | 83 | 1 |
| LobSGenPelWeeSheWor | 83 | 1 |
| EelGreGenMuHPelWeeSheShrWor | 83 | 1 |
| LobSLobFGenScSDUrDBLUHERDOG | 83 | 1 |
| LobSLobFGenScSDUrDMONGOUNDDOG | 83 | 1 |
| LobSLobFGenScSDGOUNDHERDOG | 83 | 1 |
| LobSLobFGenShrMONHERDOG | 83 | 1 |
| LobSLobFScSDMONGOUNDSKA | 83 | 1 |
| LobSLobFScSDScFDShrMON | 83 | 1 |
| LobSLobFBLUMONGOUNDRSCDOGSMB | 83 | 1 |
| LobFScFDBLUMONGOUNDRSCDOGSMBTILE | 83 | 1 |
| LobFSurFScSDScFDBLUMONGOUNDHERRSCSKADOGSMBTILE | 83 | 1 |
| LobFShrBLUMONGOUNDHERRSCSKADOGSMBTILE | 83 | 1 |
| LobSLobFShrBLUMONGOUNDHERRSCSCUSKADOGSMBTILE | 83 | 1 |
| LobSLobFGenSurFScFDBLUMONGOUNDHERSKADOGSMBTILE | 83 | 1 |
| LobSGenBSBBLUMONGOUNDDOGSMB | 83 | 1 |
| LobSGenBLUFLOGOUNDHERSKADOGSMB | 83 | 1 |
| LobSBLUMONGOUNDHERSKADOG | 83 | 1 |
| LobSLobFBLUGOUNDHERRSCDOGSMB | 83 | 1 |
| LobSGenScSDScFDShrBLUMONGOUNDDOGSMB | 83 | 1 |
| LobSLobFGenBLUGOUNDHERDOGSMB | 83 | 1 |
| LobSLobFGenScSDShrMONGOUNDDOGSMB | 83 | 1 |
| LobSLobFGenShrBLUGOUNDHERDOGSMB | 83 | 1 |
| LobSLobFScSDShrBLUMONGOUNDDOG | 83 | 1 |
| LobSLobFGenShrMONGOUNDRSCSKASMB | 83 | 1 |
| LobSLobFShrGOUNDHERRSCSKADOGSMB | 83 | 1 |
| LobSLobFGenScSDShrGOUNDRSCSKADOG | 83 | 1 |
| LobSLobFGenSurFScSDScFDShrMONHERDOGSMB | 83 | 1 |
| LobSLobFScSDScFDUrDGOUNDHERSKADOG | 83 | 1 |
| GreLobSLobFGenScSDScFDShrMONGOUNDSKATILE | 83 | 1 |
| GenSurFScSDShrMONGOUNDHERRSCSKADOGSMBTILE | 83 | 1 |
| LobSLobFShrMONGOUNDHERRSCSKADOGSMB | 83 | 1 |
| LobFGenScFDShrBLUMONGOUNDHERRSCSKADOGSMB | 78 | 1 |
| LobSGenShrBLUMONGOUNDRSCSKADOGSMBTILE | 83 | 1 |
| LobSLobFGenBLUMONGOUNDHERRSCSKADOGSMB | 83 | 1 |
| LobSLobFGenScSDShrBLUMONGOUNDHERSKADOGSMBTILE | 83 | 1 |
| LobSLobFGenShrBLUMONGOUNDHERRSCSKADOGSMBTILE | 83 | 1 |
| LobSLobFShrBLUMONGOUNDHERRSCSKADOGSMB | 83 | 1 |
| GreLobSLobFGenBLUMONGOUNDHERRSCSKADOGSMBTILE | 83 | 1 |
| LobSLobFGenBLUGOUNDRSCDOGTILE | 83 | 1 |
| LobSLobFGenBLUMONGOUNDHERRSCDOGSMBTILE | 83 | 1 |
| LobSLobFGenBLUMONGOUNDHERSKADOGSMB | 83 | 1 |
| LobSLobFGenScSDBLUMONGOUNDRSCDOG | 83 | 1 |
| LobSLobFScSDBLUGOUNDSKADOGSMBTILE | 83 | 1 |
| LobSLobFShrBLUMONGOUNDRSCDOGSMBTILE | 83 | 1 |
| LobSLobFGenQuaScSDBLUMONGOUNDHERRSCSKADOGSMBTILE | 83 | 1 |
| LobSLobFGenScSDScFDBLUMONGOUNDHERRSCSKADOGSMBTILE | 83 | 1 |
| LobSLobFGenScSDUrDBLUMONGOUNDHERRSCSKADOGSMBTILE | 83 | 1 |
| LobSLobFScSDBLUMONRSCSKADOG | 83 | 1 |
| LobSLobFScSDBLUMONRSCSKADOGSMBTILE | 83 | 1 |
| LobSLobFScSDScFDShrBLUMONRSCSKADOGSMBTILE | 83 | 1 |
| LobSScSDScFDBLUMONGOUNDHERRSCSKADOG | 83 | 1 |
| LobSGenBSBBLUFLOMONGOUNDHERSCUSKADOGSMBTILE | 83 | 1 |
| LobSLobFGenBSBBLUFLOMONGOUNDHERRSCSCUSKADOGSMB | 83 | 1 |
| LobSLobFShrBSBBLUFLOMONGOUNDHERSCUSKADOGSMBTILE | 83 | 1 |
| LobSLobFGenShrBSBBLUFLOMONGOUNDHERRSCSCUSKADOGSMBTILE | 83 | 1 |
| LobSLobFGenQuaShrBSBBLUFLOMONGOUNDHERRSCSCUSKADOGSMBTILE | 83 | 1 |
| LobSGenBSBBLUFLODOG | 83 | 1 |
| ElverLobSSheShr | 89 | 1 |
| LobSSheShrWor | 89 | 1 |
| ElverLobSScSDShe | 89 | 1 |
| PelHER | 89 | 1 |
| ElverLobSLobFGenShe | 89 | 1 |
| ElverLobSShr | 89 | 1 |
| LobSLobFWor | 89 | 1 |
| LobSShrWor | 89 | 1 |
| LobSScSDWor | 89 | 1 |
| GenScSDScHWeeUrH | 89 | 1 |
| ElverLobSShrUrH | 89 | 1 |
| LobSLobFScHShrUrH | 89 | 1 |
| GreLobSLobFGenScHShrUrH | 89 | 1 |
| ElverLobSLobFGenScHUrH | 89 | 1 |
| ElverLobSLobFUrH | 89 | 1 |
| LobSLobFSheUrH | 89 | 1 |
| LobSLobFShrUrH | 89 | 1 |
| LobSGenScSDScHSheUrH | 89 | 1 |
| LobSLobFGenScSDScHShrUrH | 89 | 1 |
| LobSLobFGenScSDUrH | 89 | 1 |
| ElverGreLobSGenScSDScHSheShrUrH | 89 | 1 |
| ElverGreGenScSDSheShrUrH | 89 | 1 |
| LobSLobFMuHScHUrHBLUGOUNDDOGSMB | 89 | 1 |
| GenPelScHShrUrHBLUGOUNDHERSKADOG | 94 | 1 |
| LobSGenMuDScSDWee | 89 | 1 |
| ElverGreLobSGenScSDSheUrD | 89 | 1 |
| ElverGreLobSScSDSheUrD | 89 | 1 |
| ElverLobSScSDSheShr | 89 | 1 |
| ElverLobSScSDUrD | 89 | 1 |
| ElverLobSMuD | 89 | 1 |
| GreGenScSDSheUrD | 89 | 1 |
| LobSGenPelScSDUrD | 89 | 1 |
| LobSSheUrD | 89 | 1 |
| GenQuaScSDSheUrD | 89 | 1 |
| GenScSDSheUrD | 89 | 1 |
| LobSGenScSDSheShrUrD | 89 | 1 |
| GenPelScSD | 89 | 1 |
| GenPelScSDSCDShrUrD | 89 | 1 |
| LobSPelScSD | 89 | 1 |
| LobSGenScSDWeeShr | 89 | 1 |
| LobSPelShr | 89 | 1 |
| GreLobSGenSheShr | 89 | 1 |
| GreLobSSheShr | 89 | 1 |
| GreLobSGenPelScSDShr | 89 | 1 |
| GreLobSGenPelShr | 89 | 1 |
| GreLobSGenScSDShe | 89 | 1 |
| ElverLobSGenPelSheShr | 89 | 1 |
| ElverLobSLobFGenPel | 89 | 1 |
| GenPelScSDSheShr | 89 | 1 |
| LobSGenMuDPelScSDSheShr | 89 | 1 |
| LobSGenPelScSDSheShr | 89 | 1 |
| LobSGenPelScSDWeeShrUrD | 89 | 1 |
| GenScSDWee | 89 | 1 |
| ElverGreGenScSDShe | 89 | 1 |
| LobSGenScSDWor | 89 | 1 |
| GreLobSGenMuHPelScSD | 89 | 1 |
| ElverEelGreLobSGenPelQuaScSDShr | 89 | 1 |
| EelLobSGenWeeSheShrWor | 89 | 1 |
| GenScSDWeeShe | 89 | 1 |
| LobSGenMuDMuHWeeShe | 89 | 1 |
| LobSGenWeeShrWor | 89 | 1 |
| GreLobSGenScSDWeeShrUrD | 89 | 1 |
| GreGenScSDWeeShrUrD | 89 | 1 |
| GreLobSLobFGenPelWee | 89 | 1 |
| LobSLobFQuaUrHHERRSCSKA | 89 | 1 |
| LobFShrUrHBLUMONGOUNDHERDOGSMB | 89 | 1 |
| LobFGenPelBLUMONGOUNDHERDOGSMBTILE | 89 | 1 |
| LobSGenScHUrHBSBBLUFLOMONGOUNDHERSCUDOGSMBTILE | 89 | 1 |
| GenPelWeeShrBLUFLOMONGOUNDHERRSCSCUSKADOGSMBTILE | 89 | 1 |
| GenPelBSBBLUFLOGOUNDHERSCUSMBTILE | 89 | 1 |
| LobSPelHERSMB | 94 | 1 |
| ElverLobSLobFSheShrGOUNDHERSMB | 94 | 1 |
| LobSLobFSheShrUrH | 94 | 1 |
| LobSLobFGenScSDSheUrH | 94 | 1 |
| LobSLobFScSDSheUrH | 94 | 1 |
| LobSLobFGenPelScSDScHUrDUrHBLUGOUNDHERSKADOGSMB | 94 | 1 |
| LobSLobFScSDSheUrD | 94 | 1 |
| LobSLobFSheUrD | 94 | 1 |
| LobSLobFGenScSDSheUrDUrHWorGOUND | 94 | 1 |
| LobSLobFGenScSDSheWor | 94 | 1 |
| LobSLobFGenScSDWor | 94 | 1 |
| ElverLobSLobFGenMuDScSD | 94 | 1 |
| ElverLobSLobFGenScSDShr | 94 | 1 |
| LobSLobFGenSheUrD | 94 | 1 |
| LobSGenPelScSDShrUrDGOUNDHER | 94 | 1 |
| LobSLobFGenPelScSD | 94 | 1 |
| LobSLobFGenPelScSDUrD | 94 | 1 |
| LobSLobFScSDSheShr | 94 | 1 |
| ElverLobSLobFScSDShr | 94 | 1 |
| LobSLobFGenPelScSDWeeShr | 94 | 1 |
| GreLobSLobFGenSheShr | 94 | 1 |
| ElverGreLobSLobFGenScSDShr | 94 | 1 |
| ElverLobSLobFGenShrUrD | 94 | 1 |
| LobSLobFGenPelGOUND | 94 | 1 |
| ElverLobSLobFGenPelShrGOUND | 94 | 1 |
| LobSLobFGenPelScSDSheShr | 94 | 1 |
| GreLobSLobFGenPelWeeShr | 94 | 1 |
| LobSLobFGenMuDPelWeeShr | 94 | 1 |
| LobSLobFGenPelScSDUrDSKADOG | 94 | 1 |
| LobSLobFGenPelScSDShrGOUNDHERDOG | 94 | 1 |
| LobSPelBLUGOUNDHERSMB | 94 | 1 |
| EelGreLobSLobFGenMuDSurSPelQuaScSDWeeShrBLUGOUNDSKADOGTILE | 94 | 1 |
| LobSGenPelShrMONGOUNDHERDOGSMB | 94 | 1 |
| LobSLobFGenPelScSDSheShrBLUMONGOUNDHERDOG | 94 | 1 |
| LobSLobFGenPelShrBLUMONGOUNDHERDOG | 94 | 1 |
| LobSLobFGenPelShrBLUMONGOUNDHERDOGSMB | 94 | 1 |
| LobSLobFGenSheShrMONGOUNDHERSKASMB | 94 | 1 |
| LobSLobFSheShrBLUFLOGOUNDHERSKASMB | 94 | 1 |
| LobSLobFGenPelScSDScFDShrUrDMONGOUNDHERRSC | 94 | 1 |
| LobSLobFGenPelShrFLOMONGOUNDRSCDOG | 94 | 1 |
| LobSGenPelShrBLUMONGOUNDSKADOGSMB | 94 | 1 |
| LobSLobFGenPelScSDScFDShrBLUMONGOUNDHERRSCSKADOGSMB | 94 | 1 |
| LobSLobFGenPelScSDShrBLUMONGOUNDHERSKADOGSMBTILE | 94 | 1 |
| LobSLobFGenPelScSDShrBLUMONGOUNDHERSKADOGTILE | 94 | 1 |
| LobSLobFGenPelShrBLUMONGOUNDRSCSKADOGSMB | 94 | 1 |
| LobSLobFGenPelShrBLUMONGOUNDHERRSCSKADOGSMB | 94 | 1 |
| LobSLobFGenPelShrBLUMONGOUNDHERRSCSKADOGSMBTILE | 94 | 1 |
| LobSLobFGenSurFPelScFDShrBLUMONGOUNDHERRSCSKADOGSMBTILE | 94 | 1 |
| LobSLobFGenSurFPelShrBLUFLOMONGOUNDHERRSCSKADOGSMB | 94 | 1 |
| LobSLobFGenSurFPelShrBLUMONGOUNDHERSKADOGSMB | 94 | 1 |
| LobSLobFGenPelBLUGOUNDHERSKADOGSMB | 94 | 1 |
| LobSLobFGenSheWorBLUMONGOUNDHERRSCSKADOGSMBTILE | 94 | 1 |
| GreLobSLobFGenScSDWeeShrBLUHERRSCSKADOGSMB | 94 | 1 |
| LobSLobFGenWeeBLUFLOMONGOUNDHERRSCSKADOGSMBTILE | 94 | 1 |
| ElverGreScSDSheBLUMONGOUNDHERRSCSKADOGSMB | 94 | 1 |
| LobSLobFScHSheUrHBLUMONGOUNDHERRSCSKADOGSMBTILE | 94 | 1 |
| GreLobSGenPelScHWeeShrUrHBLUMONGOUNDHERDOGSMBTILE | 94 | 1 |
| LobSLobFGenPelScHShrUrHBLUMONGOUNDHERRSCSKADOGSMB | 94 | 1 |
| LobSLobFGenPelScSDShrBLUFLOMONGOUNDHERSCUSKADOGSMBTILE | 94 | 1 |
| ElverGreLobSLobFWeeShrBSBBLUMONGOUNDHERSCUSKADOGSMBTILE | 94 | 1 |
