# Supplementary material for: Uneven adaptive capacity among fishers in a sea of change
Source: PLoS One. 2017 Jun 12;12(6):e0178266. doi: 10.1371/journal.pone.0178266 (PMC5467827; doi:10.1371/journal.pone.0178266)
Supplement: S2 Table — Evaluation of adaptive capacity of fishing portfolios based on the attributes identified by fisheries experts in Maine. (DOCX) [file pone.0178266.s002.docx]

**S2 Table. Adaptive capacity evaluation**

Evaluation of adaptive capacity of fishing portfolios based on the attributes identified by fisheries experts in Maine.

| **Attribute** | | **(1) Market stability** | **(2) Status of fishery** | **(3) Potential to accumulate wealth** | **(4) Existing local governance structure and industry organization(s)** | **(5) Geography** | **(6) Gear type** |
| --- | --- | --- | --- | --- | --- | --- | --- |
| **Description** | | Stable markets reduce fishermen's economic uncertainty in an otherwise dynamic and often unpredictable environment of doing business | Healthy fish stocks lead to fishing opportunities | Savings create a buffer that allow people to withstand lean periods | Participatory governance structures and self-organized fisheries organizations increase marine harvesters' ability to influence the system. | License portfolios that give harvesters access to a diverse set of geographies increase marine harvesters' flexibility. | Using different gear types allows harvesters to target a more diverse set of fisheries and increases marine harvesters' opportunities to take advantage of fisheries more consistently. |
| **Expert Perspective** | **Majority opinion** | *"We have some extraordinary examples here in Maine of markets changing so that in a given year or a given season or a given decade or time – choose your time period – that things can become far more or less lucrative depending on how the market is handling that product. Is it demanding a product that they can produce? Is it demanding it at a price point that makes sense given their costs, expenses and those sorts of things. So I think that market part is important, both in the sort of fundamental infrastructure, but also in just the evolution back through time, that that cycles, that there's all kinds of potential uncertainty there."* | *"A poor state of the fishery is bad for adaption." "I would expect more adaptation across the stable species than the one that oscillates." "What does it mean to be adaptable? Well, I think for me it means to be able to continue to do whatever it is your doing and in this instance we’re talking about fishing. So to be able to continue to do whatever it is you are doing based on the plentifulness of your species or abundance of whatever you are going after and the ability to access the fisheries, which deals directly with licensing."* | *"If you have accumulated wealth, then you can sort of make some of the investments that you need to make those transitions. You can go out and buy a federal permit. If you've got $200,000.00 sitting in the bank and you decided you wanted to go into ground fishing part time too, you could figure that out. If you don't, clearly that's not gonna be an opportunity."* | *"The communities that have non-profits or trade associations that are well established and working on some of these issues. The places where there is an ecosystem of groups that can leverage each others activities and can do work on this kind of stuff are ahead in those areas where they have those, where these have those groups working. In Maine we have a lot of groups working on the science, on the policy, and all of the coops in lobster working on marketing. I feel like as well we have really strong trade associations. We’re pretty well positioned from that perspective."* | *"I think [adaptability] means opportunity to go after different species or to use different gear types or move into different areas."* | *"At the most basic level, if you have all those skillsets and you are well-rounded, it’s probably because you have had access to or continue to have access to all of the gear types, fisheries that use all of the gear types. So meaning you’re not just a lobsterman."* |
|  | **Dissenting opinion (i.e., counter point)** | *"To some degree I feel like it's just when your back's against the wall you may be more willing to think about what your next steps are. And your option might not just be to keep going forward with your head down off the edge of the cliff, right?"* | *"To some degree I would say, in my experience, you see greater willingness to be strategic and to kind of consider yourself in a broader context or consider your fishery in a broader context when the resource is not strong."* | *"I don't know that I think that we have seen actually a lot of meaningful evidence that accumulation in a given fishery leads to better adaptability, because I think that – like people in the Elver fishery, for example, you don't see them diversifying necessarily into other fisheries. That's the only factor that allows someone to diversify, right? So does it make them more adaptable in face of any other situation in their life that might create some financial stress or something? ... I really don't know that I think accumulation of wealth is a factor in driving a lot of decisions that I see people make about their businesses."* | *"In our current management structure I don't think it's going to make you any more or less adaptive. In fact, it might make you less because you've invested so much effort and time into a single focus."* |  |  |
| **Metric** |  | We define market stability as a function of the R2 value of prices during the past 20 years (1995-2014). | We use stock assessment data from agency documents to determine the status of fisheries. In instances where stock assessments are not available we refer to recent research. Where no stock assessment data are available, we use landings over the past 5 and 20 years as a proxy for the health of each fishery by combining the direction (-/+) of the standardized regression coefficient of each time period. | We use the average value ($) of the fishery over the past 4 years (2011-2014) divided by the number of harvesters with access to the fishery. For fisheries that are harvested with state and federal fisheries the fisheries was weighted by the % of landings per sector (e.g., 80% federal vs 20% state). | We measure governance and industry organization in terms of the presence/absence of (1) a regional management structure w/ local stakeholder engagement; and (2) fisheries-specific associations. | We define geographic diversity in terms of the number of general regions (intertidal, state, and/or federal) that a fisherman can access with his/her license portfolio. | We define gear diversity in terms of the general scales of operation (hand/rake/net, hook/trap/dive, trawl/dredge/seine) that a fisherman uses to exploit a particular fishery. |
| **Data source(s)** | | MDMR (2015) | MDMR (2015), ASMFC, NEFMC, NEFCS | NOAA Fisheries (2015) | MDMR, ASMFC, NEFSC, MAFMC, GARFO | None | None |

1. Market Stability

|  | **Rank** | Score | High | Medium | Low |
| --- | --- | --- | --- | --- | --- |
| **License type** | **Species** |  | 0.5848-0.9245 | 0.2198-0.5848 | 0.0001-.2198 |
| Pelagic/Anadromous | Alewife | 0.9245 | 3 |  |  |
|  | Smelt (other) | NA |  |  |  |
|  | Atlantic menhaden (other) | NA |  |  |  |
|  | White Hake | 0.1267 |  |  | 1 |
|  | Spiny dogfish | 0.01928 |  |  | 1 |
|  | Atlantic mackerel (other) | NA |  |  |  |
|  | Long finned squid (other) | NA |  |  |  |
|  | Short finned squid (other) | NA |  |  |  |
|  | Butterfish (other) | NA |  |  |  |
|  | Scup (other) | NA |  |  |  |
|  | Black sea bass (other) | NA |  |  |  |
|  | Atlantic Herring | 0.9212 | 3 |  |  |
|  | Average |  |  |  |  |
| MusselH | Blue mussel (whole) | 0.5264 |  | 2 |  |
| MusselD | Blue mussel (whole) | 0.5264 |  | 2 |  |
| SpinyDogfish | Spiny dogfish | 0.01928 |  |  | 1 |
| Eel | Eel | 0.6432 | 3 |  |  |
| Elver | Elver | 0.3898 |  | 2 |  |
| SurfS | Hard clam | 0.2999 |  | 2 |  |
| SurfF | Hard clam | 0.2999 |  | 2 |  |
| Herring | Atlantic Herring | 0.9212 | 3 |  |  |
| LobsterS | American Lobster | 0.03051 |  | 2 |  |
| LobsterF | American Lobster | 0.03051 |  | 2 |  |
|  | Crab (all) | 0.2453 |  | 2 |  |
| RedSeaCrab | Red Crab | NA |  | 2 |  |
| GreenCrab | GreenCrab (other) | NA |  | 2 |  |
| QuahogD | Mahogany Quahog | 0.01004 |  |  | 1 |
| Monkfish | Monkfish | 0.5192 |  | 2 |  |
| General | Periwinkles | 0.6742 | 3 |  |  |
|  | Atlantic Halibut | 0.723 | 3 |  |  |
|  | Atlantic Hagfish (other) | NA |  |  |  |
|  | Whelk (other) | NA |  |  |  |
|  | Skate | 0.06275 |  |  | 1 |
|  | Atlantic Cod | 0.7092 | 3 |  |  |
|  | American Plaice | 0.2626 |  | 2 |  |
|  | Atlantic Pollock | 0.1296 |  |  | 1 |
|  | Acadian Redfish | 0.1006 |  |  | 1 |
|  | Bluefish (other) | NA |  |  |  |
|  | Cusk | 0.13 |  |  | 1 |
|  | Haddock | 0.116 |  |  | 1 |
|  | White Hake | 0.1267 |  |  | 1 |
|  | Wolffish | 0.1943 |  |  | 1 |
|  | Winter Flounder | 0.307 |  | 2 |  |
|  | Witch Flounder | 0.3531 |  | 2 |  |
|  | Yellowtail Flounder | 0.003508 |  |  | 1 |
|  | Tilefish (other) | NA |  |  |  |
|  | Sea raven (other) | NA |  |  |  |
|  | Average |  |  |  |  |
| Worm | Sandworm | 0.858 | 3 |  |  |
|  | Bloodworm | 0.7673 | 3 |  |  |
|  | Average |  |  |  |  |
| SeaCucumberD | Sea cucumber | 0.6555 | 3 |  |  |
| ScallopSD | Scallop | 0.5162 | 3 |  |  |
| ScallopFD | Scallop | 0.5162 | 3 |  |  |
| ScallopH | Scallop | 0.5162 | 3 |  |  |
| Seaweed | Seaweed | 0.2835 |  | 2 |  |
| Shrimp | Northern Shrimp | 0.0001065 |  |  | 1 |
| Skate | Skate | 0.06275 |  |  | 1 |
| Shellfish | Softshell clam | 0.7203 | 3 |  |  |
|  | Atlantic razor clam (other) | NA |  |  |  |
| UrchinD | Urchin | 0.8183 | 3 |  |  |
| UrchinH | Urchin | 0.8183 | 3 |  |  |
| Groundfish | Atlantic Cod | 0.7092 | 3 |  |  |
|  | American Plaice | 0.2626 |  | 2 |  |
|  | Atlantic Pollock | 0.1296 |  |  | 1 |
|  | Acadian Redfish | 0.1006 |  |  | 1 |
|  | Cusk | 0.13 |  |  | 1 |
|  | Haddock | 0.116 |  |  | 1 |
|  | White Hake | 0.1267 |  |  | 1 |
|  | Wolffish | 0.1943 |  |  | 1 |
|  | Average |  |  |  |  |
| Flounder | Winter Flounder | 0.307 |  | 2 |  |
|  | Witch Flounder | 0.3531 |  | 2 |  |
|  | Yellowtail Flounder | 0.003508 |  |  | 1 |
|  | Average |  |  |  |  |
| HighlyMigSpecies | Swordfish (other) | NA |  |  |  |
|  | Porbeagle shark (other) | NA |  |  |  |
|  | Mako shortfinned shark (other) | NA |  |  |  |
|  | Bluefin tuna | NA |  |  |  |
|  | Escolar (other) | NA |  |  |  |
|  | Amberjack (other) | NA |  |  |  |
|  |  |  |  |  |  |
| BlackSeaBass |  | NA |  |  |  |
| Bluefish | Bluefish (other) | NA |  |  |  |
| Scup |  | NA |  |  |  |
| SquidMackButter | Atlantic mackerel (other) | NA |  |  |  |
|  | Long finned squid (other) | NA |  |  |  |
|  | Short finned squid (other) | NA |  |  |  |
|  | Butterfish (other) | NA |  |  |  |
| Tilefish | Golden tilefish (other) | NA |  |  |  |
|  | Average | 0.35761375 |  |  |  |
|  | Classificaiton method | Used Fisher-Jenks natural break calculation to determine classification | | | |

2. Status of Fishery

|  | **Rank** | Score | | | High | Medium | Low |
| --- | --- | --- | --- | --- | --- | --- | --- |
| **License type** | **Species** | 20 Yrs | 5 Yrs | Status |  |  |  |
| Pelagic/Anadromous | Alewife | 0.8424632 | 0.7194692 | The 2012 river herring benchmark stock assessment found of the 52 stocks of alewife and blueback herring for which data were available for use in the assessment, 23 were depleted relative to historic levels, one stock was increasing, and the status of 28 stocks could not be determined because the time-series of available data was too short. Estimates of abundance and fishing mortality could not be developed because of the lack of adequate data. The depleted determination was used instead of overfished because of the many factors that have contributed to the declining abundance of river herring, which include not just directed and incidental fishing, but also habitat loss, predation, and climate change (http://www.asmfc.org/species/shad-river-herring). |  |  | 1 |
|  | Smelt (other) | NA |  | In 2004, the National Oceanic and Atmospheric Administration listed the rainbow smelt as a federal Species of Concern. The rainbow smelt is a small fish that lives in estuaries and offshore waters, and spawns in shallow freshwater streams each spring. Its numbers have dropped dramatically during the last fifteen to twenty years for reasons that are not well understood (http://www.maine.gov/dmr/smelt/). |  |  | 1 |
|  | Atlantic menhaden (other) | NA |  | Based on the revised reference points recommended by the benhcmark stock assessment and approved by the Peer Review Panel, Atlantic menhaden are neither overfished nor experiencing overfishing. The revised reference points are based on historical performance of the population during the time frame 1960-2012, a period during which the Technical Committee considers the population to have been sustainably fished. Fishing mortality rates have remained below the revised overfishing threshold (1.26) since the 1960s, and have hovered around the revised overfishing target (0.38) through the 1990s. In 2003, fishing mortality dropped below the revised target and was estimated to be 0.22 in 2013 (the latest year in the assessment). In other words, fishing mortality has been decreasing throughout the history of the fishery, and is now 42% below the target (http://www.asmfc.org/species/atlantic-menhaden). | 3 |  |  |
|  | White Hake | -0.7621461 | 0.4682099 | Based on this updated assessment, white hake (Urophycis tenuis) stock is not over shed and over shing is not occurring Based on this updated assessment, white hake (Urophycis tenuis) stock is not over fished and over fishing is not occurring (http://www.nefsc.noaa.gov/publications/crd/crd1524/Individual%20Stocks/White_hake.pdf). | 3 |  |  |
|  | Spiny dogfish | -0.5624532 | -0.4972446 | The revised 2015 stock assessment update indicates spiny dogfish are not overfished and not experiencing overfishing. Spawning stock biomass is estimated to be at 106% of the target (http://www.asmfc.org/species/spiny-dogfish). | 3 |  |  |
|  | Atlantic mackerel (other) | NA |  | Unknown |  | 2 |  |
|  | Long finned squid (other) | NA |  | Unknown |  | 2 |  |
|  | Short finned squid (other) | NA |  | Unknown |  | 2 |  |
|  | Butterfish (other) | NA |  | No overfishing and the stock is not overfished (http://www.greateratlantic.fisheries.noaa.gov/sustainable/species/msb/) | 3 |  |  |
|  | Scup (other) | NA |  | Scup was declared rebuilt  ahead of schedule in 2009 after a benchmark stock assessment determined that the stock was no longer overfished and overfishing was not occurring (http://static1.squarespace.com/static/511cdc7fe4b00307a2628ac6/t/557afd7be4b03c2094a30e39/1434123643450/Scup_fishery_info_doc2015_final.pdf) . | 3 |  |  |
|  | Black sea bass (other) | NA |  | The 2012 stock assessment update indicates that black sea bass is continues to be rebuilt; it is not overfished and is experiencing overfishing. | 3 |  |  |
|  | Atlantic Herring | -0.3227807 | 0.8082424 | The latest stock assessment update, conducted by the Northeast Regional Stock Assessment Workshop in 2015, indicates Atlantic herring are not overfished and overfishing is not occurring. Spawning stock biomass in 2014 is estimated at 623,000 mt, well above the SSB threshold and target of 155,573 mt (342 million pounds) and 311,145 mt (685 million pounds), respectively. Current fishing mortality is estimated at 0.16, below the fishing mortality threshold of 0.24 (http://www.asmfc.org/species/atlantic-herring) | 3 |  |  |
|  | Average |  |  |  |  |  |  |
| MusselH | Blue mussel (whole) | -0.6600729 | -0.8006693 | Unknown (http://www.nmfs.noaa.gov/pr/interactions/fisheries/table2/GME_mussel_dredge.html) |  | 2 |  |
| MusselD | Blue mussel (whole) | -0.6600729 | -0.8006693 | Unknown (http://www.nmfs.noaa.gov/pr/interactions/fisheries/table2/GME_mussel_dredge.html) |  | 2 |  |
| SpinyDogfish | Spiny dogfish | -0.5624532 | -0.4972446 | The revised 2015 stock assessment update indicates spiny dogfish are not overfished and not experiencing overfishing. Spawning stock biomass is estimated to be at 106% of the target (http://www.asmfc.org/species/spiny-dogfish). | 3 |  |  |
| Eel | Eel | -0.7855394 | 0.5545367 | According to the 2012 benchmark stock assessment, American eel population is depleted in U.S. waters. The stock is at or near historically low levels due to a combination of historical overfishing, habitat loss, food web alterations, predation, turbine mortality, environmental changes, toxins and contaminants, and disease (http://www.asmfc.org/species/american-eel) |  |  | 1 |
| Elver | Elver | 0.1388093 | 0.4763302 | According to the 2012 benchmark stock assessment, American eel population is depleted in U.S. waters. The stock is at or near historically low levels due to a combination of historical overfishing, habitat loss, food web alterations, predation, turbine mortality, environmental changes, toxins and contaminants, and disease (http://www.asmfc.org/species/american-eel) |  |  | 1 |
| SurfS | Hard clam | 0.5121653 | -0.6010235 | The Atlantic surfclam resource in the US EEZ is not overfished and overfishing is not  occurring in 2011 (http://nefsc.noaa.gov/publications/crd/crd1304/parta.pdf). | 3 |  |  |
| SurfF | Hard clam | 0.5121653 | -0.6010235 | The Atlantic surfclam resource in the US EEZ is not overfished and overfishing is not  occurring in 2011 (http://nefsc.noaa.gov/publications/crd/crd1304/parta.pdf). | 3 |  |  |
| Herring | Atlantic Herring | -0.3227807 | 0.8082424 | The latest stock assessment update, conducted by the Northeast Regional Stock Assessment Workshop in 2015, indicates Atlantic herring are not overfished and overfishing is not occurring. Spawning stock biomass in 2014 is estimated at 623,000 mt, well above the SSB threshold and target of 155,573 mt (342 million pounds) and 311,145 mt (685 million pounds), respectively. Current fishing mortality is estimated at 0.16, below the fishing mortality threshold of 0.24 (http://www.asmfc.org/species/atlantic-herring) | 3 |  |  |
| LobsterS | American Lobster | 0.9310941 | 0.8487512 | The 2015 American Lobster Benchmark Stock Assessment and Peer Review Report indicates the American lobster resource presents a mixed picture of stock status, with record high stock abundance and recruitment in the Gulf of Maine (GOM) and Georges Bank (GBK), and record low abundance and recruitment in Southern New England (SNE)(http://www.asmfc.org/species/american-lobster) | 3 |  |  |
| LobsterF | American Lobster | 0.9310941 | 0.8487512 | The 2015 American Lobster Benchmark Stock Assessment and Peer Review Report indicates the American lobster resource presents a mixed picture of stock status, with record high stock abundance and recruitment in the Gulf of Maine (GOM) and Georges Bank (GBK), and record low abundance and recruitment in Southern New England (SNE)(http://www.asmfc.org/species/american-lobster) | 3 |  |  |
|  | Crab (all) | NA | NA | Multiple species (Unknown) |  |  | 1 |
| RedSeaCrab | Red Crab | NA | NA | The red crab fishery was certified by the Marine Stewardship Council as sustainable in the fall of 2009 (http://www.greateratlantic.fisheries.noaa.gov/sustainable/species/redcrab/). |  |  | 1 |
| GreenCrab | GreenCrab (other) | NA | NA |  |  |  | 1 |
| QuahogD | Mahogany Quahog | -0.3282133 | -0.6446383 | No overfishing and stock is not overfished (http://www.seagrant.umaine.edu/maine-seafood-guide/hard-clams),(http://www.nefsc.noaa.gov/publications/crd/crd0710/pdfs/appa8.pdf) | 3 |  |  |
| Monkfish | Monkfish | -0.7923824 | 0.4684363 | Monkfish in both the northern and  southern management areas are not overfished and overfishing is not occurring (http://archive.nefmc.org/monk/). | 3 |  |  |
| General | Periwinkles | 0.5448914 | -0.8485708 | Unknown (http://www.maine.gov/dmr/rm/whelks.html) |  | 2 |  |
|  | Atlantic Halibut | 0.7907239 | 0.9654393 | Based on this updated assessment, Atlantic halibut ( Hippoglossus hippoglossus) stock status is unknown (Figures 81-82)(http://www.nefsc.noaa.gov/publications/crd/crd1524/Individual%20Stocks/Atlantic_halibut.pdf). |  | 2 |  |
|  | Atlantic Hagfish (other) | NA | NA | NA |  |  |  |
|  | Whelk (other) | NA | NA | Unknown (http://www.maine.gov/dmr/rm/whelks.html) |  | 2 |  |
|  | Skate | -0.9193246 | NA | Based on the reference points  developed at SAW 30 and the proposed overfishing definitions for each skate species, barndoor skate and thorny skate are currently considered to be in an overfished condition. At the time that SAW 30 was conducted, overfishing was thought to be occurring on winter skate. Fishing  mortality estimates for winter skate are not considered to be reliable, so it is not possible to  determine whether or not overfishing is still occurring on winter skate (http://s3.amazonaws.com/nefmc.org/FINAL.Skate-FMP.EIS.FINAL.VOL.I.pdf). |  |  | 1 |
|  | Atlantic Cod | -0.9023167 | -0.8537472 | Based on this updated assessment, the Gulf of Maine Atlantic cod (Gadus morhua) stock is over fished and over fishing is occurring (http://www.nefsc.noaa.gov/publications/crd/crd1524/Individual%20Stocks/GOM_Atlantic_cod.pdf) |  |  | 1 |
|  | American Plaice | -0.8830361 | 0.8998854 | The Gulf of Maine – Georges Bank American plaice stock is not overfished and overfishing is  not occurring (http://www.nefsc.noaa.gov/publications/crd/crd1206/americanplaice.pdf). | 3 |  |  |
|  | Atlantic Pollock | -0.3722108 | 0.5336328 | Comparing the current 2009 estimates of SSB and F to the MSY reference points, the stock is not overfished and overfishing is not occurring. | 3 |  |  |
|  | Acadian Redfish | 0.2335976 | 0.4113396 | Based on this updated assessment, the Acadian red sh ( Sebastes fasciatus) stock is not over shed and over shing is not occurring ( | 3 |  |  |
|  | Bluefish (other) | NA | NA | Based on the 2015 benchmark stock assessment and peer review conducted by the Northeast Regional Stock Assessment Workshop, bluefish are not overfished and not experiencing overfishing relative to the new biological reference points defined in the assessment (http://www.asmfc.org/species/bluefish). | 3 |  |  |
|  | Cusk | -0.8230537 | NA | Cusk (Brosme brosme) are a National Marine Fisheries Service (NMFS) "species of concern," as well as a "candidate species" under the Endangered Species Act (ESA) as we are currently conducting a status review on the species (http://www.greateratlantic.fisheries.noaa.gov/protected/pcp/soc/cusk.html). |  |  | 1 |
|  | Haddock | -0.4277021 | 0.6630766 | Comparing the current 2009 estimates of SSB and F to the MSY reference points, the stock is not overfished and overfishing is not occurring (http://www.nefsc.noaa.gov/publications/crd/crd1017/pdfs/pollock.pdf). | 3 |  |  |
|  | White Hake | -0.7621461 | 0.4682099 | Based on this updated assessment, white hake (Urophycis tenuis) stock is not over shed and over shing is not occurring Based on this updated assessment, white hake (Urophycis tenuis) stock is not over shed and over shing is not occurring (http://www.nefsc.noaa.gov/publications/crd/crd1524/Individual%20Stocks/White_hake.pdf). | 3 |  |  |
|  | Wolffish | -0.9564943 | NA | Based on this updatedassessment, the Atlantic wol sh (Anarhichas lupus) stock is over shed and over shing is not occurring (http://www.nefsc.noaa.gov/publications/crd/crd1524/Individual%20Stocks/Atlantic_wolffish.pdf). |  |  | 1 |
|  | Winter Flounder | 0.5158058 | NA | Based on this updated assessment, the Gulf of Maine winter ounder (Pseudo- pleuronectes americanus) stock biomass status is unknown and over shing is not occurring (http://www.nefsc.noaa.gov/publications/crd/crd1524/Individual%20Stocks/GOM_winter_flounder.pdf). |  | 2 |  |
|  | Witch Flounder | -0.8955651 | 0.2630926 | The 2010 spawning stock biomass was 4,099 mt, 41% below SSBmsy (10,051 mt) and 2010 fishing mortality was 0.47, 173% above Fmsy (F=0.27); therefore, witch flounder was overfished and  overfishing occurred in 2010. |  |  | 1 |
|  | Yellowtail Flounder | -0.2792362 | NA | Based on this updated assessment, Cape Cod-Gulf of Maine yellowtail ounder (Limanda ferruginea) stock is over shed and over shing is occurring (http://www.nefsc.noaa.gov/publications/crd/crd1524/Individual%20Stocks/CCGoM_yellowtail_flounder.pdf). |  |  | 1 |
|  | Tilefish (other) | NA | NA | NA |  |  |  |
|  | Sea raven (other) | NA | NA | NA |  |  |  |
|  | Average |  |  |  |  |  |  |
| Worm | Sandworm | 0.1860569 | -0.7911157 | NA |  | 2 |  |
|  | Bloodworm | 0.269166 | -0.8214759 | NA |  | 2 |  |
|  | Average |  |  |  |  |  |  |
| SeaCucumberD | Sea cucumber | 0.1572108 | NA | Stock biomass decreased substantially from 2005 to 2006, but was stable from 2006 to 2007 (http://www.maine.gov/dmr/cukes/chen2007.pdf). |  |  | 1 |
| ScallopSD | Scallop | -0.6185465 | 0.9481895 | Overall, both catch and effort have declined during the ten years that the DMR has collected landings data, while prices have increased (http://www.maine.gov/dmr/cukes/feindeletal2011.pdf). Since 2007, Maine fishermen & the Department of Marine Resources (DMR) have implemented a series of forward thinking management measures aimed at rebuilding the depleted scallop resource. The fishery has begun to realize the benefits of these measures, as recent reports indicate landings in 2013 alone were the highest in 13 years (424,547 meat pounds) while the value of the fishery was the highest in 15 years ($5,194,553) (http://www.maine.gov/dmr/rm/scallops/management/index.htm). | 3 |  |  |
| ScallopFD | Scallop | -0.6185465 | 0.9481895 | Overall, both catch and effort have declined during the ten years that the DMR has collected landings data, while prices have increased (http://www.maine.gov/dmr/cukes/feindeletal2011.pdf). Since 2007, Maine fishermen & the Department of Marine Resources (DMR) have implemented a series of forward thinking management measures aimed at rebuilding the depleted scallop resource. The fishery has begun to realize the benefits of these measures, as recent reports indicate landings in 2013 alone were the highest in 13 years (424,547 meat pounds) while the value of the fishery was the highest in 15 years ($5,194,553) (http://www.maine.gov/dmr/rm/scallops/management/index.htm). | 3 |  |  |
| ScallopH | Scallop | -0.6185465 | 0.9481895 | Overall, both catch and effort have declined during the ten years that the DMR has collected landings data, while prices have increased (http://www.maine.gov/dmr/cukes/feindeletal2011.pdf). Since 2007, Maine fishermen & the Department of Marine Resources (DMR) have implemented a series of forward thinking management measures aimed at rebuilding the depleted scallop resource. The fishery has begun to realize the benefits of these measures, as recent reports indicate landings in 2013 alone were the highest in 13 years (424,547 meat pounds) while the value of the fishery was the highest in 15 years ($5,194,553) (http://www.maine.gov/dmr/rm/scallops/management/index.htm). | 3 |  |  |
| Seaweed | Seaweed | 0.9541144 | 0.9657694 | Multiple species (Unknown) |  | 2 |  |
| Shrimp | Northern Shrimp | -0.3693034 | -0.9751001 | Since the 2014 benchmark stock assessment was not accepted by the peer review panel for management use, the Northern Shrimp Technical Committee evaluated a suite of indicators to determine the status of the stock for 2015. Using these indicators, the Technical Committee found abundance and biomass indices for 2012-2015 were the lowest on record for the 32-year time series. The stock has experience failed recruitment for five consecutive years, including the three smallest year classes on record. As a result, the indices of fishable biomass from 2012-2015 are the lowest on record (http://www.asmfc.org/species/northern-shrimp). |  |  | 1 |
| Skate | Skate | -0.9193246 | NA | Based on the reference points  developed at SAW 30 and the proposed overfishing definitions for each skate species, barndoor skate and thorny skate are currently considered to be in an overfished condition. At the time that SAW 30 was conducted, overfishing was thought to be occurring on winter skate. Fishing  mortality estimates for winter skate are not considered to be reliable, so it is not possible to  determine whether or not overfishing is still occurring on winter skate (http://s3.amazonaws.com/nefmc.org/FINAL.Skate-FMP.EIS.FINAL.VOL.I.pdf). |  |  | 1 |
| Shellfish | Softshell clam | 0.1202588 | -0.2706821 |  |  | 2 |  |
|  | Atlantic razor clam (other) | NA | NA |  |  |  |  |
| UrchinD | Urchin | -0.8609714 | -0.8406917 | http://www.maine.gov/dmr/rm/seaurchin/datareport2015.pdf |  |  | 1 |
| UrchinH | Urchin | -0.8609714 | -0.8406917 | http://www.maine.gov/dmr/rm/seaurchin/datareport2015.pdf |  |  | 1 |
| Groundfish | Atlantic Cod | -0.9023167 | -0.8537472 | Based on this updated assessment, the Gulf of Maine Atlantic cod (Gadus morhua) stock is over shed and over shing is occurring (http://www.nefsc.noaa.gov/publications/crd/crd1524/Individual%20Stocks/GOM_Atlantic_cod.pdf) |  |  | 1 |
|  | American Plaice | -0.8830361 | 0.8998854 | The Gulf of Maine – Georges Bank American plaice stock is not overfished and overfishing is  not occurring (http://www.nefsc.noaa.gov/publications/crd/crd1206/americanplaice.pdf). | 3 |  |  |
|  | Atlantic Pollock | -0.3722108 | 0.5336328 | Comparing the current 2009 estimates of SSB and F to the MSY reference points, the stock is not overfished and overfishing is not occurring. | 3 |  |  |
|  | Acadian Redfish | 0.2335976 | 0.4113396 | Based on this updated assessment, the Acadian red sh ( Sebastes fasciatus) stock is not over shed and over shing is not occurring ( | 3 |  |  |
|  | Cusk | -0.8230537 | NA | Cusk (Brosme brosme) are a National Marine Fisheries Service (NMFS) "species of concern," as well as a "candidate species" under the Endangered Species Act (ESA) as we are currently conducting a status review on the species (http://www.greateratlantic.fisheries.noaa.gov/protected/pcp/soc/cusk.html). |  |  | 1 |
|  | Haddock | -0.4277021 | 0.6630766 | Comparing the current 2009 estimates of SSB and F to the MSY reference points, the stock is not overfished and overfishing is not occurring (http://www.nefsc.noaa.gov/publications/crd/crd1017/pdfs/pollock.pdf). | 3 |  |  |
|  | White Hake | -0.7621461 | 0.4682099 | Based on this updated assessment, white hake (Urophycis tenuis) stock is not over shed and over shing is not occurring Based on this updated assessment, white hake (Urophycis tenuis) stock is not over shed and over shing is not occurring (http://www.nefsc.noaa.gov/publications/crd/crd1524/Individual%20Stocks/White_hake.pdf). | 3 |  |  |
|  | Wolffish | -0.9564943 | NA | Based on this updatedassessment, the Atlantic wol sh (Anarhichas lupus) stock is over shed and over shing is not occurring (http://www.nefsc.noaa.gov/publications/crd/crd1524/Individual%20Stocks/Atlantic_wolffish.pdf). |  |  | 1 |
|  | Average |  |  |  |  |  |  |
| Flounder | Winter Flounder | 0.5158058 | NA | Based on this updated assessment, the Gulf of Maine winter ounder (Pseudo- pleuronectes americanus) stock biomass status is unknown and over shing is not occurring (http://www.nefsc.noaa.gov/publications/crd/crd1524/Individual%20Stocks/GOM_winter_flounder.pdf). |  | 2 |  |
|  | Witch Flounder | -0.8955651 | 0.2630926 | The 2010 spawning stock biomass was 4,099 mt, 41% below SSBmsy (10,051 mt) and 2010 fishing mortality was 0.47, 173% above Fmsy (F=0.27); therefore, witch flounder was overfished and  overfishing occurred in 2010. |  |  | 1 |
|  | Yellowtail Flounder | -0.2792362 | NA | Based on this updated assessment, Cape Cod-Gulf of Maine yellowtail ounder (Limanda ferruginea) stock is over shed and over shing is occurring (http://www.nefsc.noaa.gov/publications/crd/crd1524/Individual%20Stocks/CCGoM_yellowtail_flounder.pdf). |  |  | 1 |
|  | Average |  |  |  |  |  |  |
| HighlyMigSpecies | Swordfish (other) | NA | NA | Not overfished and no overfishing (http://www.iccat.int/Documents/Meetings/Docs/2009_SWO_ASSESS_ENG.pdf) | 3 |  |  |
|  | Porbeagle shark (other) | NA | NA | Species of concern (http://www.nmfs.noaa.gov/pr/pdfs/species/porbeagleshark_detailed.pdf) |  |  | 1 |
|  | Mako shortfinned shark (other) | NA | NA | IUCN listed (http://www.iucnredlist.org/details/39341/0) |  |  | 1 |
|  | Bluefin tuna | NA | NA | Species of concern (http://www.fisheries.noaa.gov/pr/pdfs/species/bluefintuna_highlights.pdf) |  |  | 1 |
|  | Escolar (other) | NA | NA | Unknown |  | 2 |  |
|  | Amberjack (other) | NA | NA | According to the 2008 stock assessment, South Atlantic greater amberjack are not overfished, and are not subject to overfishing (http://sedarweb.org/docs/sar/S15%20SAR2%20Greater%20Amberjack%20FINAL.pdf) | 3 |  |  |
|  |  |  |  |  |  |  |  |
| BlackSeaBass |  | NA | NA | When the assessment model was updated in 2012, it was determined that the stock was not overfished and that overfishing was not occurring in 2011 (http://static1.squarespace.com/static/511cdc7fe4b00307a2628ac6/t/557f2a3ee4b06c23617fe385/1434397246767/BSB_fishery_info_doc2015_final.pdf) | 3 |  |  |
| Bluefish | Bluefish (other) | NA | NA | Based on the 2015 benchmark stock assessment and peer review conducted by the Northeast Regional Stock Assessment Workshop, bluefish are not overfished and not experiencing overfishing relative to the new biological reference points defined in the assessment (http://www.asmfc.org/species/bluefish). | 3 |  |  |
| Scup |  | NA | NA | Scup was declared rebuilt  ahead of schedule in 2009 after a benchmark stock assessment determined that the stock was no longer overfished and overfishing was not occurring (http://static1.squarespace.com/static/511cdc7fe4b00307a2628ac6/t/557afd7be4b03c2094a30e39/1434123643450/Scup_fishery_info_doc2015_final.pdf) . | 3 |  |  |
| SquidMackButter | Atlantic mackerel (other) | NA | NA | Unknown |  | 2 |  |
|  | Long finned squid (other) | NA | NA | Unknown |  | 2 |  |
|  | Short finned squid (other) | NA | NA | Unknown |  | 2 |  |
|  | Butterfish (other) | NA | NA | No overfishing and the stock is not overfished (http://www.greateratlantic.fisheries.noaa.gov/sustainable/species/msb/) | 3 |  |  |
| Tilefish | Golden tilefish (other) | NA | NA |  |  |  |  |
|  | Average |  |  |  |  |  |  |
|  | Classificaiton method | Stock status based on agency data. If no data, than we used landings trends as proxys whereas if coefficient is: (+, +) = High; (+, -) = Medium; (-, -) = Low | | | | | |

3. Potential to accumulate wealth

|  | **Rank** | Score |  | High | Medium | Low |
| --- | --- | --- | --- | --- | --- | --- |
| **License type** | **Species** | Average $ | # Licenses | 125491-32709 | 32709-7951 | 7951-0 |
| Pelagic/Anadromous | Alewife | $2,099.13 | 185 |  |  | 1 |
|  | Smelt (other) | NA | 185 |  |  | 1 |
|  | Atlantic menhaden (other) | NA | 185 |  |  | 1 |
|  | White Hake | $0.00 | 185 |  |  | 1 |
|  | Spiny dogfish | $1.43 | 185 |  |  | 1 |
|  | Atlantic mackerel (other) | NA | 185 |  |  | 1 |
|  | Long finned squid (other) | NA | 185 |  |  | 1 |
|  | Short finned squid (other) | NA | 185 |  |  | 1 |
|  | Butterfish (other) | NA | 185 |  |  | 1 |
|  | Scup (other) | NA | 185 |  |  | 1 |
|  | Black sea bass (other) | NA | 185 |  |  | 1 |
|  | Atlantic Herring | $0.00 | 185 |  |  | 1 |
|  | Average |  |  |  |  |  |
| MusselH | Blue mussel (whole) | $46,425.68 | 45 | 3 |  |  |
| MusselD | Blue mussel (whole) | $46,425.68 | 45 | 3 |  |  |
| SpinyDogfish | Spiny dogfish | $186.01 | 227 |  |  | 1 |
| Eel | Eel | $653.44 | 36 |  |  | 1 |
| Elver | Elver | $47,572.30 | 470 | 3 |  |  |
| SurfS | Hard clam | $15,921.53 | 32 |  | 2 |  |
| SurfF | Hard clam | $15,921.53 | 32 |  | 2 |  |
| Herring | Atlantic Herring | $78,198.76 | 187 | 3 |  |  |
| LobsterS | American Lobster | $43,005.73 | 4969 | 3 |  |  |
| LobsterF | American Lobster | $125,490.71 | 1296 | 3 |  |  |
|  | Crab (all) | $2,167.91 | 357 |  |  | 1 |
| RedSeaCrab | Red Crab | NA | 117 |  |  | 1 |
| GreenCrab | GreenCrab (other) | NA | 243 |  |  | 1 |
| QuahogD | Mahogany Quahog | $59,911.44 | 27 | 3 |  |  |
| Monkfish | Monkfish | $3,726.98 | 195 |  |  | 1 |
| General | Periwinkles | $503.94 | 1667 |  |  | 1 |
|  | Atlantic Halibut | $200.86 | 1667 |  |  | 1 |
|  | Atlantic Hagfish (other) | NA | 1667 |  |  | 1 |
|  | Whelk (other) | NA | 1667 |  |  | 1 |
|  | Skate | $0.00 | 1667 |  |  | 1 |
|  | Atlantic Cod | $15.76 | 1667 |  |  | 1 |
|  | American Plaice | $0.00 | 1667 |  |  | 1 |
|  | Atlantic Pollock | $40.78 | 1667 |  |  | 1 |
|  | Acadian Redfish | $1.74 | 1667 |  |  | 1 |
|  | Bluefish (other) | NA | 1667 |  |  | 1 |
|  | Cusk | $0.00 | 1667 |  |  | 1 |
|  | Haddock | $0.00 | 1667 |  |  | 1 |
|  | White Hake | $0.10 | 1667 |  |  | 1 |
|  | Wolffish | $0.00 | 1667 |  |  | 1 |
|  | Winter Flounder | $0.00 | 1667 |  |  | 1 |
|  | Witch Flounder | $4.25 | 1667 |  |  | 1 |
|  | Yellowtail Flounder | $0.00 | 1667 |  |  | 1 |
|  | Tilefish (other) | NA | 1667 |  |  | 1 |
|  | Sea raven (other) | NA | 1667 |  |  | 1 |
|  | Average |  |  |  |  |  |
| Worm | Sandworm | $1,588.42 | 853 |  |  | 1 |
|  | Bloodworm | $6,783.50 | 853 |  |  | 1 |
|  | Average |  |  |  |  |  |
| SeaCucumberD | Sea cucumber | $6,603.36 | 9 |  |  | 1 |
| ScallopSD | Scallop | $59,638.68 | 612 | 3 |  |  |
| ScallopFD | Scallop | $22,411.36 | 49 |  | 2 |  |
| ScallopH | Scallop | $59,638.68 | 612 | 3 |  |  |
| Seaweed | Seaweed | $4,135.91 | 157 |  |  | 1 |
| Shrimp | Northern Shrimp | $6,289.91 | 524 |  |  | 1 |
| Skate | Skate | $0.00 | 166 |  |  | 1 |
| Shellfish | Softshell clam | $9,119.32 | 1893 |  | 2 |  |
|  | Atlantic razor clam (other) | NA | NA |  |  | 1 |
| UrchinD | Urchin | $58,518.21 | 295 | 3 |  |  |
| UrchinH | Urchin | $58,518.21 | 295 | 3 |  |  |
| Groundfish | Atlantic Cod | $4,246.92 | 257 |  |  | 1 |
|  | American Plaice | $2,232.52 | 257 |  |  | 1 |
|  | Atlantic Pollock | $9,362.51 | 257 |  | 2 |  |
|  | Acadian Redfish | $1,253.82 | 257 |  |  | 1 |
|  | Cusk | $68.22 | 257 |  |  | 1 |
|  | Haddock | $711.91 | 257 |  |  | 1 |
|  | White Hake | $1,745.86 | 257 |  |  | 1 |
|  | Wolffish | $0.00 | 257 |  |  | 1 |
|  | Average |  |  |  |  |  |
| Flounder | Winter Flounder | $0.00 | 64 |  |  | 1 |
|  | Witch Flounder | $6,432.63 | 64 |  |  | 1 |
|  | Yellowtail Flounder | $0.00 | 64 |  |  | 1 |
|  | Average |  |  |  |  |  |
| HighlyMigSpecies | Swordfish (other) | NA | NA |  |  | 1 |
|  | Porbeagle shark (other) | NA | NA |  |  | 1 |
|  | Mako shortfinned shark (other) | NA | NA |  |  | 1 |
|  | Bluefin tuna | $15,630.01 | 33 |  | 2 |  |
|  | Escolar (other) | NA | NA |  |  | 1 |
|  | Amberjack (other) | NA | NA |  |  | 1 |
|  |  |  |  |  |  |  |
| BlackSeaBass |  | NA | 45 |  |  | 1 |
| Bluefish | Bluefish (other) | NA | 213 |  |  | 1 |
| Scup |  | NA | 49 |  |  | 1 |
| SquidMackButter | Atlantic mackerel (other) | NA | 203 |  |  | 1 |
|  | Long finned squid (other) | NA | 203 |  |  | 1 |
|  | Short finned squid (other) | NA | 203 |  |  | 1 |
|  | Butterfish (other) | NA | 203 |  |  | 1 |
| Tilefish | Golden tilefish (other) | NA | 124 |  |  | 1 |
|  | Average |  |  |  |  |  |
|  | Classificaiton method | Used Fisher-Jenks natural break calculation to determine classification, including the outlier for federal lobster fisheries. Note: federal lobster was assigned a score of 3. | | | | |

4. Existing local governance structure and industry organization(s)

|  | **Rank** | Score | | High | Medium | Low |
| --- | --- | --- | --- | --- | --- | --- |
| **License type** | **Species** | Local management unit | Fishery association |  |  |  |
| Pelagic/Anadromous | Alewife | Y (Alewife Management Plans) (http://www.maine.gov/dmr/searunfish/alewife/) | Y (Alewife Harvesters of Maine)(2010) https://www.facebook.com/AlewifeHarvestersOfMaine/ | 3 |  |  |
|  | Smelt (other) | N | N |  |  | 1 |
|  | Atlantic menhaden (other) | N | N |  |  | 1 |
|  | White Hake | N | N |  |  | 1 |
|  | Spiny dogfish | N | N |  |  | 1 |
|  | Atlantic mackerel (other) | N | N |  |  | 1 |
|  | Long finned squid (other) | N | N |  |  | 1 |
|  | Short finned squid (other) | N | N |  |  | 1 |
|  | Butterfish (other) | N | N |  |  | 1 |
|  | Scup (other) | N | N |  |  | 1 |
|  | Black sea bass (other) | N | N |  |  | 1 |
|  | Atlantic Herring | N | N (East Coast Pelagic Association) (2002)(Dissolved)https://icrs.informe.org/nei-sos-icrs/ICRS?CorpSumm=20020574ND |  |  | 1 |
|  | Average |  |  |  |  |  |
| MusselH | Blue mussel (whole) | N | N |  |  | 1 |
| MusselD | Blue mussel (whole) | N | N |  |  | 1 |
| SpinyDogfish | Spiny dogfish | N | N |  |  | 1 |
| Eel | Eel | N | N |  |  | 1 |
| Elver | Elver | N | Y (Maine Elver Association) https://www.facebook.com/Maine-Elver-Fishermen-Association-166147253582461/info/?tab=page_info, American Eel Sustainability Association (http://americaneel.org/) |  |  |  |
| SurfS | Hard clam | N | N |  |  | 1 |
| SurfF | Hard clam | N | N |  |  | 1 |
| Herring | Atlantic Herring | N | N (East Coast Pelagic Association) (2002)(Dissolved)https://icrs.informe.org/nei-sos-icrs/ICRS?CorpSumm=20020574ND |  |  | 1 |
| LobsterS | American Lobster | Y (Lobster Zone Councils) | Y (Maine Lobstermen's Association)(1990)(Downeast Lobstermen's Association)(1991) Island Fishermen's Wives' Association (1994) (Southern Maine Lobstermen's Association (1998) (Maine Lobstering Union)(2013)(Maine Lobster Marketing Collaborative (numerous cooperatives), Maine Lobster Advisory Council | 3 |  |  |
| LobsterF | American Lobster | Y (Lobster Zone Councils) | Y (Maine Lobstermen's Association)(1990)(Downeast Lobstermen's Association)(1991) Island Fishermen's Wives' Association (1994) (Southern Maine Lobstermen's Association (1998) (Maine Lobstering Union)(2013)(Maine Lobster Marketing Collaborative (numerous cooperatives) | 3 |  |  |
|  | Crab (all) | N | N |  |  | 1 |
| RedSeaCrab | Red Crab | N | N |  |  | 1 |
| GreenCrab | GreenCrab (other) | N | N |  |  | 1 |
| QuahogD | Mahogany Quahog | N | N |  |  | 1 |
| Monkfish | Monkfish | N | N |  |  | 1 |
| General | Periwinkles | N | N |  |  | 1 |
|  | Atlantic Halibut | N | N |  |  | 1 |
|  | Atlantic Hagfish (other) | N | N |  |  | 1 |
|  | Whelk (other) | N | N |  |  | 1 |
|  | Skate | N | N |  |  | 1 |
|  | Atlantic Cod | N | Y (Maine Coast Fishermen's Association) (http://www.mainecoastfishermen.org/)Maine Coast Community Sector (2010)(http://www.mainecoastfishermen.org/#!sector/hej26)Maine Fishermen's Wives Association (1988)(Dissolved) https://www.google.com/search?q=maine+monkfish+association&ie=utf-8&oe=utf-8#q=maine+fishermen%27s+wives+association, Northeast Coastal Communities Sector (2010) http://www.penobscoteast.org/programs/northeast-coastal-communities-sector/ Northeast Seafood Coalition (http://northeastseafoodcoalition.org/) |  | 2 |  |
|  | American Plaice | N | Y (Maine Coast Fishermen's Association) (http://www.mainecoastfishermen.org/)Maine Coast Community Sector (2010)(http://www.mainecoastfishermen.org/#!sector/hej26)Maine Fishermen's Wives Association (1988)(Dissolved) https://www.google.com/search?q=maine+monkfish+association&ie=utf-8&oe=utf-8#q=maine+fishermen%27s+wives+association, Northeast Coastal Communities Sector (2010) http://www.penobscoteast.org/programs/northeast-coastal-communities-sector/ Northeast Seafood Coalition (http://northeastseafoodcoalition.org/) |  | 2 |  |
|  | Atlantic Pollock | N | Y (Maine Coast Fishermen's Association) (http://www.mainecoastfishermen.org/)Maine Coast Community Sector (2010)(http://www.mainecoastfishermen.org/#!sector/hej26)Maine Fishermen's Wives Association (1988)(Dissolved) https://www.google.com/search?q=maine+monkfish+association&ie=utf-8&oe=utf-8#q=maine+fishermen%27s+wives+association, Northeast Coastal Communities Sector (2010) http://www.penobscoteast.org/programs/northeast-coastal-communities-sector/ Northeast Seafood Coalition (http://northeastseafoodcoalition.org/) |  | 2 |  |
|  | Acadian Redfish | N | Y (Maine Coast Fishermen's Association) (http://www.mainecoastfishermen.org/)Maine Coast Community Sector (2010)(http://www.mainecoastfishermen.org/#!sector/hej26)Maine Fishermen's Wives Association (1988)(Dissolved) https://www.google.com/search?q=maine+monkfish+association&ie=utf-8&oe=utf-8#q=maine+fishermen%27s+wives+association, Northeast Coastal Communities Sector (2010) http://www.penobscoteast.org/programs/northeast-coastal-communities-sector/ Northeast Seafood Coalition (http://northeastseafoodcoalition.org/) |  | 2 |  |
|  | Bluefish (other) | N | Y (Maine Coast Fishermen's Association) (http://www.mainecoastfishermen.org/)Maine Coast Community Sector (2010)(http://www.mainecoastfishermen.org/#!sector/hej26)Maine Fishermen's Wives Association (1988)(Dissolved) https://www.google.com/search?q=maine+monkfish+association&ie=utf-8&oe=utf-8#q=maine+fishermen%27s+wives+association, Northeast Coastal Communities Sector (2010) http://www.penobscoteast.org/programs/northeast-coastal-communities-sector/ Northeast Seafood Coalition (http://northeastseafoodcoalition.org/) |  | 2 |  |
|  | Cusk | N | Y (Maine Coast Fishermen's Association) (http://www.mainecoastfishermen.org/)Maine Coast Community Sector (2010)(http://www.mainecoastfishermen.org/#!sector/hej26)Maine Fishermen's Wives Association (1988)(Dissolved) https://www.google.com/search?q=maine+monkfish+association&ie=utf-8&oe=utf-8#q=maine+fishermen%27s+wives+association, Northeast Coastal Communities Sector (2010) http://www.penobscoteast.org/programs/northeast-coastal-communities-sector/ Northeast Seafood Coalition (http://northeastseafoodcoalition.org/) |  | 2 |  |
|  | Haddock | N | Y (Maine Coast Fishermen's Association) (http://www.mainecoastfishermen.org/)Maine Coast Community Sector (2010)(http://www.mainecoastfishermen.org/#!sector/hej26)Maine Fishermen's Wives Association (1988)(Dissolved) https://www.google.com/search?q=maine+monkfish+association&ie=utf-8&oe=utf-8#q=maine+fishermen%27s+wives+association, Northeast Coastal Communities Sector (2010) http://www.penobscoteast.org/programs/northeast-coastal-communities-sector/ Northeast Seafood Coalition (http://northeastseafoodcoalition.org/) |  | 2 |  |
|  | White Hake | N | Y (Maine Coast Fishermen's Association) (http://www.mainecoastfishermen.org/)Maine Coast Community Sector (2010)(http://www.mainecoastfishermen.org/#!sector/hej26)Maine Fishermen's Wives Association (1988)(Dissolved) https://www.google.com/search?q=maine+monkfish+association&ie=utf-8&oe=utf-8#q=maine+fishermen%27s+wives+association, Northeast Coastal Communities Sector (2010) http://www.penobscoteast.org/programs/northeast-coastal-communities-sector/ Northeast Seafood Coalition (http://northeastseafoodcoalition.org/) |  | 2 |  |
|  | Wolffish | N | Y (Maine Coast Fishermen's Association) (http://www.mainecoastfishermen.org/)Maine Coast Community Sector (2010)(http://www.mainecoastfishermen.org/#!sector/hej26)Maine Fishermen's Wives Association (1988)(Dissolved) https://www.google.com/search?q=maine+monkfish+association&ie=utf-8&oe=utf-8#q=maine+fishermen%27s+wives+association, Northeast Coastal Communities Sector (2010) http://www.penobscoteast.org/programs/northeast-coastal-communities-sector/ Northeast Seafood Coalition (http://northeastseafoodcoalition.org/) |  | 2 |  |
|  | Winter Flounder | N | Y (Maine Coast Fishermen's Association) (http://www.mainecoastfishermen.org/)Maine Coast Community Sector (2010)(http://www.mainecoastfishermen.org/#!sector/hej26)Maine Fishermen's Wives Association (1988)(Dissolved) https://www.google.com/search?q=maine+monkfish+association&ie=utf-8&oe=utf-8#q=maine+fishermen%27s+wives+association, Northeast Coastal Communities Sector (2010) http://www.penobscoteast.org/programs/northeast-coastal-communities-sector/ Northeast Seafood Coalition (http://northeastseafoodcoalition.org/) |  | 2 |  |
|  | Witch Flounder | N | Y (Maine Coast Fishermen's Association) (http://www.mainecoastfishermen.org/)Maine Coast Community Sector (2010)(http://www.mainecoastfishermen.org/#!sector/hej26)Maine Fishermen's Wives Association (1988)(Dissolved) https://www.google.com/search?q=maine+monkfish+association&ie=utf-8&oe=utf-8#q=maine+fishermen%27s+wives+association, Northeast Coastal Communities Sector (2010) http://www.penobscoteast.org/programs/northeast-coastal-communities-sector/ Northeast Seafood Coalition (http://northeastseafoodcoalition.org/) |  | 2 |  |
|  | Yellowtail Flounder | N | Y (Maine Coast Fishermen's Association) (http://www.mainecoastfishermen.org/)Maine Coast Community Sector (2010)(http://www.mainecoastfishermen.org/#!sector/hej26)Maine Fishermen's Wives Association (1988)(Dissolved) https://www.google.com/search?q=maine+monkfish+association&ie=utf-8&oe=utf-8#q=maine+fishermen%27s+wives+association, Northeast Coastal Communities Sector (2010) http://www.penobscoteast.org/programs/northeast-coastal-communities-sector/ Northeast Seafood Coalition (http://northeastseafoodcoalition.org/) |  | 2 |  |
|  | Tilefish (other) | N | N |  |  | 1 |
|  | Sea raven (other) | N | N |  |  | 1 |
|  | Average |  |  |  |  |  |
| Worm | Sandworm | N | Y (Maine Worm Harvester's Association)(2014) https://www.facebook.com/Independent-Maine-Marine-Worm-Harvesters-Association-1516334565292022/ |  | 2 |  |
|  | Bloodworm | N | Y (Maine Worm Harvester's Association)(2014) https://www.facebook.com/Independent-Maine-Marine-Worm-Harvesters-Association-1516334565292022/ |  | 2 |  |
|  | Average |  |  |  |  |  |
| SeaCucumberD | Sea cucumber | N | N |  |  | 1 |
| ScallopSD | Scallop | Y Scallop Advisory Council (http://www.maine.gov/dmr/council/scallops/index.htm) | N |  | 2 |  |
| ScallopFD | Scallop | N | Y (Eastern New England Scallop Association) (http://enescallop.com/) |  | 2 |  |
| ScallopH | Scallop | Y Scallop Advisory Council (http://www.maine.gov/dmr/council/scallops/index.htm) | N |  | 2 |  |
| Seaweed | Seaweed | Y (Rodkweed Fishery Management Plan Development Team) (2013) http://www.maine.gov/dmr/rm/rockweed/pdt.htm, Rockweed Working Group (http://www.maine.gov/dmr/council/index.htm) | N |  | 2 |  |
| Shrimp | Northern Shrimp | N | N |  |  | 1 |
| Skate | Skate | N | N |  |  | 1 |
| Shellfish | Softshell clam | Y (Shellfish Advisory Council)http://www.maine.gov/dmr/council/shellfish/index.htm, Maine Shellfish Management Programs | Y (Maine Clammers Assocation) (2008) http://maineclammers.org/ | 3 |  |  |
|  | Atlantic razor clam (other) | N | N (Marine Urchin Harvester's Association) (Established in 1994 but dissolved) https://icrs.informe.org/nei-sos-icrs/ICRS?CorpSumm=19940257ND |  |  | 1 |
| UrchinD | Urchin | Y (Urchin Advisory Council) http://www.maine.gov/dmr/council/sea_urchin/index.htm | N (Marine Urchin Harvester's Association) (Established in 1994 but dissolved) https://icrs.informe.org/nei-sos-icrs/ICRS?CorpSumm=19940257ND |  | 2 |  |
| UrchinH | Urchin | Y (Urchin Advisory Council) http://www.maine.gov/dmr/council/sea_urchin/index.htm | N |  | 2 |  |
| Groundfish | Atlantic Cod | N | Y (Maine Coast Fishermen's Association) (http://www.mainecoastfishermen.org/)Maine Coast Community Sector (2010)(http://www.mainecoastfishermen.org/#!sector/hej26)Maine Fishermen's Wives Association (1988)(Dissolved) https://www.google.com/search?q=maine+monkfish+association&ie=utf-8&oe=utf-8#q=maine+fishermen%27s+wives+association, Northeast Coastal Communities Sector (2010) http://www.penobscoteast.org/programs/northeast-coastal-communities-sector/ Northeast Seafood Coalition (http://northeastseafoodcoalition.org/) |  | 2 |  |
|  | American Plaice | N | Y (Maine Coast Fishermen's Association) (http://www.mainecoastfishermen.org/)Maine Coast Community Sector (2010)(http://www.mainecoastfishermen.org/#!sector/hej26)Maine Fishermen's Wives Association (1988)(Dissolved) https://www.google.com/search?q=maine+monkfish+association&ie=utf-8&oe=utf-8#q=maine+fishermen%27s+wives+association, Northeast Coastal Communities Sector (2010) http://www.penobscoteast.org/programs/northeast-coastal-communities-sector/ Northeast Seafood Coalition (http://northeastseafoodcoalition.org/) |  | 2 |  |
|  | Atlantic Pollock | N | Y (Maine Coast Fishermen's Association) (http://www.mainecoastfishermen.org/)Maine Coast Community Sector (2010)(http://www.mainecoastfishermen.org/#!sector/hej26)Maine Fishermen's Wives Association (1988)(Dissolved) https://www.google.com/search?q=maine+monkfish+association&ie=utf-8&oe=utf-8#q=maine+fishermen%27s+wives+association, Northeast Coastal Communities Sector (2010) http://www.penobscoteast.org/programs/northeast-coastal-communities-sector/ Northeast Seafood Coalition (http://northeastseafoodcoalition.org/) |  | 2 |  |
|  | Acadian Redfish | N | Y (Maine Coast Fishermen's Association) (http://www.mainecoastfishermen.org/)Maine Coast Community Sector (2010)(http://www.mainecoastfishermen.org/#!sector/hej26)Maine Fishermen's Wives Association (1988)(Dissolved) https://www.google.com/search?q=maine+monkfish+association&ie=utf-8&oe=utf-8#q=maine+fishermen%27s+wives+association, Northeast Coastal Communities Sector (2010) http://www.penobscoteast.org/programs/northeast-coastal-communities-sector/ Northeast Seafood Coalition (http://northeastseafoodcoalition.org/) |  | 2 |  |
|  | Cusk | N | Y (Maine Coast Fishermen's Association) (http://www.mainecoastfishermen.org/)Maine Coast Community Sector (2010)(http://www.mainecoastfishermen.org/#!sector/hej26)Maine Fishermen's Wives Association (1988)(Dissolved) https://www.google.com/search?q=maine+monkfish+association&ie=utf-8&oe=utf-8#q=maine+fishermen%27s+wives+association, Northeast Coastal Communities Sector (2010) http://www.penobscoteast.org/programs/northeast-coastal-communities-sector/ Northeast Seafood Coalition (http://northeastseafoodcoalition.org/) |  | 2 |  |
|  | Haddock | N | Y (Maine Coast Fishermen's Association) (http://www.mainecoastfishermen.org/)Maine Coast Community Sector (2010)(http://www.mainecoastfishermen.org/#!sector/hej26)Maine Fishermen's Wives Association (1988)(Dissolved) https://www.google.com/search?q=maine+monkfish+association&ie=utf-8&oe=utf-8#q=maine+fishermen%27s+wives+association, Northeast Coastal Communities Sector (2010) http://www.penobscoteast.org/programs/northeast-coastal-communities-sector/ Northeast Seafood Coalition (http://northeastseafoodcoalition.org/) |  | 2 |  |
|  | White Hake | N | Y (Maine Coast Fishermen's Association) (http://www.mainecoastfishermen.org/)Maine Coast Community Sector (2010)(http://www.mainecoastfishermen.org/#!sector/hej26)Maine Fishermen's Wives Association (1988)(Dissolved) https://www.google.com/search?q=maine+monkfish+association&ie=utf-8&oe=utf-8#q=maine+fishermen%27s+wives+association, Northeast Coastal Communities Sector (2010) http://www.penobscoteast.org/programs/northeast-coastal-communities-sector/ Northeast Seafood Coalition (http://northeastseafoodcoalition.org/) |  | 2 |  |
|  | Wolffish | N | Y (Maine Coast Fishermen's Association) (http://www.mainecoastfishermen.org/)Maine Coast Community Sector (2010)(http://www.mainecoastfishermen.org/#!sector/hej26)Maine Fishermen's Wives Association (1988)(Dissolved) https://www.google.com/search?q=maine+monkfish+association&ie=utf-8&oe=utf-8#q=maine+fishermen%27s+wives+association, Northeast Coastal Communities Sector (2010) http://www.penobscoteast.org/programs/northeast-coastal-communities-sector/ Northeast Seafood Coalition (http://northeastseafoodcoalition.org/) |  | 2 |  |
|  | Average |  |  |  |  |  |
| Flounder | Winter Flounder | N | Y (Maine Coast Fishermen's Association) (http://www.mainecoastfishermen.org/)Maine Coast Community Sector (2010)(http://www.mainecoastfishermen.org/#!sector/hej26)Maine Fishermen's Wives Association (1988)(Dissolved) https://www.google.com/search?q=maine+monkfish+association&ie=utf-8&oe=utf-8#q=maine+fishermen%27s+wives+association, Northeast Coastal Communities Sector (2010) http://www.penobscoteast.org/programs/northeast-coastal-communities-sector/ Northeast Seafood Coalition (http://northeastseafoodcoalition.org/) |  | 2 |  |
|  | Witch Flounder | N | Y (Maine Coast Fishermen's Association) (http://www.mainecoastfishermen.org/)Maine Coast Community Sector (2010)(http://www.mainecoastfishermen.org/#!sector/hej26)Maine Fishermen's Wives Association (1988)(Dissolved) https://www.google.com/search?q=maine+monkfish+association&ie=utf-8&oe=utf-8#q=maine+fishermen%27s+wives+association, Northeast Coastal Communities Sector (2010) http://www.penobscoteast.org/programs/northeast-coastal-communities-sector/ Northeast Seafood Coalition (http://northeastseafoodcoalition.org/) |  | 2 |  |
|  | Yellowtail Flounder | N | Y (Maine Coast Fishermen's Association) (http://www.mainecoastfishermen.org/)Maine Coast Community Sector (2010)(http://www.mainecoastfishermen.org/#!sector/hej26)Maine Fishermen's Wives Association (1988)(Dissolved) https://www.google.com/search?q=maine+monkfish+association&ie=utf-8&oe=utf-8#q=maine+fishermen%27s+wives+association, Northeast Coastal Communities Sector (2010) http://www.penobscoteast.org/programs/northeast-coastal-communities-sector/ Northeast Seafood Coalition (http://northeastseafoodcoalition.org/) |  | 2 |  |
|  | Average |  |  |  |  |  |
| HighlyMigSpecies | Swordfish (other) | N | N |  |  | 1 |
|  | Porbeagle shark (other) | N | N |  |  | 1 |
|  | Mako shortfinned shark (other) | N | N |  |  | 1 |
|  | Bluefin tuna | N | N |  |  | 1 |
|  | Escolar (other) | N | N |  |  | 1 |
|  | Amberjack (other) | N | N |  |  | 1 |
|  |  |  |  |  |  |  |
| BlackSeaBass |  | N | N |  |  | 1 |
| Bluefish | Bluefish (other) | N | N |  |  | 1 |
| Scup |  | N | N |  |  | 1 |
| SquidMackButter | Atlantic mackerel (other) | N | N |  |  | 1 |
|  | Long finned squid (other) | N | N |  |  | 1 |
|  | Short finned squid (other) | N | N |  |  | 1 |
|  | Butterfish (other) | N | N |  |  | 1 |
| Tilefish | Golden tilefish (other) | N | N |  |  | 1 |
|  | Average |  |  |  |  |  |
|  | Classificaiton method |  |  |  |  |  |

5/6. Geographic and gear diversity

|  | **Rank** | Intertidal | State | Federal | Hand | Hook/Trap | Trawl |
| --- | --- | --- | --- | --- | --- | --- | --- |
| **License type** | **Species** |  |  |  |  |  |  |
| Pelagic/Anadromous | Alewife | 1 |  |  | 1 |  |  |
|  | Smelt (other) | 1 |  |  | 1 |  |  |
|  | Atlantic menhaden (other) |  | 1 |  |  |  | 1 |
|  | White Hake |  | 1 |  |  | 1 |  |
|  | Spiny dogfish |  | 1 |  |  | 1 |  |
|  | Atlantic mackerel (other) |  | 1 |  |  | 1 |  |
|  | Long finned squid (other) |  | 1 |  |  | 1 |  |
|  | Short finned squid (other) |  | 1 |  |  | 1 |  |
|  | Butterfish (other) |  | 1 |  |  | 1 |  |
|  | Scup (other) |  | 1 |  |  | 1 |  |
|  | Black sea bass (other) |  | 1 |  |  | 1 |  |
|  | Atlantic Herring |  | 1 |  | 1 |  |  |
|  | Average |  |  |  |  |  |  |
| MusselH | Blue mussel (whole) |  | 1 |  | 1 |  |  |
| MusselD | Blue mussel (whole) |  | 1 |  |  |  | 1 |
| SpinyDogfish | Spiny dogfish |  | 1 |  |  | 1 |  |
| Eel | Eel |  | 1 |  |  | 1 |  |
| Elver | Elver | 1 |  |  | 1 |  |  |
| SurfS | Hard clam |  | 1 |  |  |  | 1 |
| SurfF | Hard clam |  |  | 1 |  |  | 1 |
| Herring | Atlantic Herring |  |  | 1 |  |  | 1 |
| LobsterS | American Lobster |  | 1 |  |  | 1 |  |
| LobsterF | American Lobster |  |  | 1 |  |  | 1 |
|  | Crab (all) |  | 1 |  |  | 1 |  |
| RedSeaCrab | Red Crab |  |  | 1 |  | 1 |  |
| GreenCrab | GreenCrab (other) |  | 1 |  |  | 1 |  |
| QuahogD | Mahogany Quahog |  | 1 |  |  |  | 1 |
| Monkfish | Monkfish |  | 1 |  |  | 1 |  |
| General | Periwinkles | 1 |  |  | 1 |  |  |
|  | Atlantic Halibut |  | 1 |  |  | 1 |  |
|  | Atlantic Hagfish (other) |  | 1 |  |  | 1 |  |
|  | Whelk (other) |  | 1 |  |  | 1 |  |
|  | Skate |  | 1 |  |  | 1 |  |
|  | Atlantic Cod |  | 1 |  |  | 1 |  |
|  | American Plaice |  | 1 |  |  | 1 |  |
|  | Atlantic Pollock |  | 1 |  |  | 1 |  |
|  | Acadian Redfish |  | 1 |  |  | 1 |  |
|  | Bluefish (other) |  | 1 |  |  | 1 |  |
|  | Cusk |  | 1 |  |  | 1 |  |
|  | Haddock |  | 1 |  |  | 1 |  |
|  | White Hake |  | 1 |  |  | 1 |  |
|  | Wolffish |  | 1 |  |  | 1 |  |
|  | Winter Flounder |  | 1 |  |  | 1 |  |
|  | Witch Flounder |  | 1 |  |  | 1 |  |
|  | Yellowtail Flounder |  | 1 |  |  | 1 |  |
|  | Tilefish (other) |  | 1 |  |  | 1 |  |
|  | Sea raven (other) |  | 1 |  |  | 1 |  |
|  | Average |  |  |  |  |  |  |
| Worm | Sandworm | 1 |  |  | 1 |  |  |
|  | Bloodworm | 1 |  |  | 1 |  |  |
|  | Average |  |  |  |  |  |  |
| SeaCucumberD | Sea cucumber |  | 1 |  |  |  | 1 |
| ScallopSD | Scallop |  | 1 |  |  |  | 1 |
| ScallopFD | Scallop |  |  | 1 |  |  | 1 |
| ScallopH | Scallop |  | 1 |  |  | 1 |  |
| Seaweed | Seaweed | 1 |  |  | 1 |  |  |
| Shrimp | Northern Shrimp |  | 1 |  |  | 1 | 1 |
| Skate | Skate |  | 1 |  |  |  | 1 |
| Shellfish | Softshell clam | 1 |  |  | 1 |  |  |
|  | Atlantic razor clam (other) | 1 |  |  | 1 |  |  |
| UrchinD | Urchin |  | 1 |  |  |  | 1 |
| UrchinH | Urchin |  | 1 |  |  | 1 |  |
| Groundfish | Atlantic Cod |  |  | 1 |  |  | 1 |
|  | American Plaice |  |  | 1 |  |  | 1 |
|  | Atlantic Pollock |  |  | 1 |  |  | 1 |
|  | Acadian Redfish |  |  | 1 |  |  | 1 |
|  | Cusk |  |  | 1 |  |  | 1 |
|  | Haddock |  |  | 1 |  |  | 1 |
|  | White Hake |  |  | 1 |  |  | 1 |
|  | Wolffish |  |  | 1 |  |  | 1 |
|  | Average |  |  |  |  |  |  |
| Flounder | Winter Flounder |  |  | 1 |  |  | 1 |
|  | Witch Flounder |  |  | 1 |  |  | 1 |
|  | Yellowtail Flounder |  |  | 1 |  |  | 1 |
|  | Average |  |  |  |  |  |  |
| HighlyMigSpecies | Swordfish (other) |  |  | 1 |  | 1 |  |
|  | Porbeagle shark (other) |  |  | 1 |  | 1 |  |
|  | Mako shortfinned shark (other) |  |  | 1 |  | 1 |  |
|  | Bluefin tuna |  |  | 1 |  | 1 |  |
|  | Escolar (other) |  |  | 1 |  | 1 |  |
|  | Amberjack (other) |  |  | 1 |  | 1 |  |
|  |  |  |  |  |  |  |  |
| BlackSeaBass |  |  |  | 1 |  | 1 |  |
| Bluefish | Bluefish (other) |  |  | 1 |  |  | 1 |
| Scup |  |  |  | 1 |  |  | 1 |
| SquidMackButter | Atlantic mackerel (other) |  |  | 1 |  |  | 1 |
|  | Long finned squid (other) |  |  | 1 |  |  | 1 |
|  | Short finned squid (other) |  |  | 1 |  |  | 1 |
|  | Butterfish (other) |  |  | 1 |  |  | 1 |
| Tilefish | Golden tilefish (other) |  |  | 1 |  | 1 |  |
